# Janus Dendrimers to Assess the Anti-HCV Activity of Molecules in Cell-Assays

**DOI:** 10.3390/pharmaceutics12111062

**Published:** 2020-11-07

**Authors:** María San Anselmo, Alexandre Lancelot, Julia E. Egido, Rafael Clavería-Gimeno, Álvaro Casanova, José Luis Serrano, Silvia Hernández-Ainsa, Olga Abian, Teresa Sierra

**Affiliations:** 1 Instituto de Nanociencia y Materiales de Aragón (INMA), University of Zaragoza-CSIC, Pedro Cerbuna 12, 50009 Zaragoza, Spain; msananselmo@unizar.es (M.S.A.); alexandrelancelot@gmail.com (A.L.); J.EgidoEgido@umcutrecht.nl (J.E.E.); joseluis@unizar.es (J.L.S.); 2 Instituto Aragonés de Ciencias de la Salud (IACS), 50009 Zaragoza, Spain; rafacg@bifi.es; 3 Institute of Biocomputation and Physics of Complex Systems (BIFI), Joint Unit IQFR-CSIC-BIFI, Universidad de Zaragoza, 50018 Zaragoza, Spain; 4 Aragon Institute for Health Research (IIS Aragon), 50009 Zaragoza, Spain; 5 Departamento de Farmacología y Fisiología, Facultad de Medicina, Universidad de Zaragoza, 50009 Zaragoza, Spain; alvarocasanov@gmail.com; 6 ARAID Foundation, Government of Aragón, 50018 Zaragoza, Spain; 7 Centro de Investigación Biomédica en Red en el Área Temática de Enfermedades Hepáticas y Digestivas (CIBERehd), 28029 Barcelona, Spain; 8 Departamento de Bioquímica y Biología Molecular y Celular, Universidad de Zaragoza, 50009 Zaragoza, Spain

**Keywords:** dendrimers, micellar aggregates, self-assembly, drug delivery, hepatitis C, antiviral drug

## Abstract

The use of nanocarriers has been revealed as a valid strategy to facilitate drug bioavailability, and this allows for expanding the drug libraries for the treatment of certain diseases such as viral diseases. In the case of Hepatitis C, the compounds iopanoic acid and 3,3′,5-triiodothyroacetic acid (or tiratricol) were identified in a primary screening as bioactive allosteric inhibitors of viral NS3 protease, but they did not exhibit accurate activity inhibiting viral replication in cell-based assays. In this work, dendritic nanocarriers are proposed due to their unique properties as drug delivery systems to rescue the bioactivity of these two drugs. Specifically, four different amphiphilic Janus dendrimers synthesized by combining 2,2′-*bis*(hydroxymethyl)propionic acid (*bis*-MPA) and 2,2′-*bis*(glyciloxy)propionic acid (*bis*-GMPA) functionalized with either hydrophilic or lipophilic moieties at their periphery were used to entrap iopanoic acid and tiratricol. Interestingly, differences were found in the loading efficiencies depending on the dendrimer design, which also led to morphological changes of the resulting dendrimer aggregates. The most remarkable results consist of the increased water solubility of the bioactive compounds within the dendrimers and the improved antiviral activity of some of the dendrimer/drug aggregates, considerably improving antiviral activity in comparison to the free drugs. Moreover, imaging studies have been developed in order to elucidate the mechanism of cellular internalization.

## 1. Introduction

The main concerns with conventional therapies against infectious diseases are related to the viability of infected cells and the development of resistances. Although the use of combination therapies could be a solution in some cases, the discovery of effective drugs is still a need. In this sense, high-throughput screening methodologies focused on determining the interactions between the isolated target biomolecule and a potential drug constitute a helpful primary screening tool to identify potential drugs among a great amount of molecules [1,2,3].

**Figure 1:**
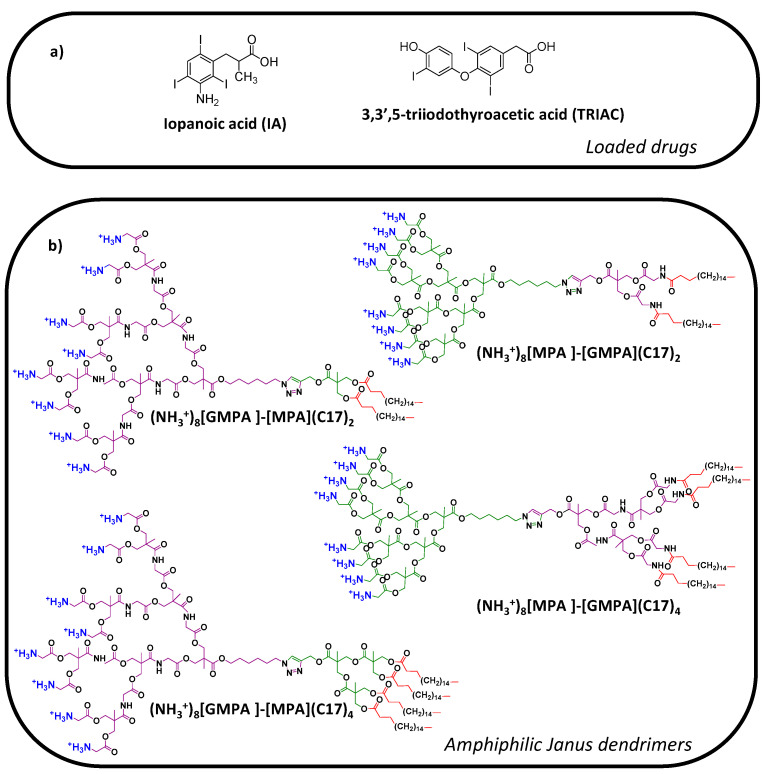
(**a**) Chemical structure of the drugs studied for anti-HCV therapy; (**b**) chemical structure of the amphiphilic Janus dendrimers.

Different strategies such as drug repurposing [4] or the application of mathematical models [5] have been revealed as valid strategies to identify new and effective antivirals or a combination of them. In the case of Hepatitis C, the identification of the HCV NS3 protease as a therapeutic target led to the application of a primary screening strategy to well-known compounds approved by FDA initially designed for other therapeutic purposes [6]. A screening with a battery of compounds against partially folded NS3 allowed for the selection of a library of molecules that stabilize this inactive conformation. Among them, iopanoic acid (IA) and 3,3′,5-triiodothyroacetic acid (or tiratricol, TRIAC), both used for thyroid-related diseases, were identified as potent bioactive allosteric inhibitors of the HCV NS3 protease (Figure 1a). However, during the secondary screening based on cell assays of inhibition of viral replication, IA and TRIAC did not exhibit appropriate inhibiting activity in cell-based assays [6], likely due to cell internalization difficulties of these compounds. Far from invalidating the therapeutic possibilities of such drugs, these drawbacks fostered the search for alternative delivery strategies. In this respect, nanocarrier-based drug delivery arises as a promising approach to rescue the bioactivity observed in primary screenings [7], as it can provide a positive input on drug pharmacokinetics favouring drug bioavailability, drug targeting, cellular internalization, and drug activity/response [8,9,10].

In the search for suitable drug nanocarriers that could permit broadening the possibilities to treat viral infections revisiting drugs with promising activity, dendrimer-based nanocarriers have emerged as alluring synthetic systems because of their unique properties including controlled architecture, versatile functionalization and cargo, and the transport and release of diverse molecules of interest [11,12]. The architecture and chemical structure of the dendritic scaffold together with its terminal groups defines the interaction with cargo molecules and their efficient uptake by the host cells. As for delivery biomedical applications, dendrimers have been extensively investigated as drug and DNA carriers for cancer-related biomedical applications [13]. Nevertheless, there is intense research ongoing in the field of infectious diseases, where the urgent implementation of strategies for the prevention and cure is taking advantage of dendrimers as carriers for different cargos as antigens [14,15] or drugs [16,17].

The versatile self-assembly behavior of amphiphilic Janus dendrimers has turned into a powerful approach to prepare nanocarriers [18,19,20], which have been shown to be advantageous for drug-delivery applications [21,22,23,24]. Our group previously reported amphiphilic Janus dendrimers based on 2,2′-*bis*(hydroxymethyl)propionic acid (*bis*-MPA) dendrons of different generations that were useful for the delivery of anti-HCV drugs. Those Janus dendrimers were composed by a hydrophilic dendron terminated in amino groups and a lipophilic dendron functionalized with stearic acid chains. Suitable combinations of the generation numbers of the hydrophilic and lipophilic dendrons provided carriers that solubilized camptothecin (CPT) used as an anti-HCV drug, and showed a higher therapeutic effect than the free drug, although at low CPT doses does increasing its therapeutic index [25]. In our search for more efficient dendritic nanostructures to carry anti-HCV drugs, we anticipated the interest of differentiating not only the terminal functionalization of each dendron, i.e., amino and stearic acid groups, but also their chemical structure. For this purpose, we selected the poly(esteramide) structure built from the 2,2′-*bis*(glyciloxy)propionic acid (*bis*-GMPA) monomer, which incorporates inner amide groups that could bring additional possibilities of interactions with cargo molecules. In fact, *bis*-GMPA dendrimers resulted in effective nanocarriers for CPT, less cytotoxic than free CPT [26].

Accordingly, we present here four novel amphiphilic Janus dendrimers thoughtfully designed, in which four regions with different chemical features can be identified. Specifically, as shown in Figure 1b these amphiphilic Janus dendrimers **(NH_3_^+^)_8_[GMPA]-[MPA](C17)_2_, (NH_3_^+^)_8_[GMPA]-[MPA](C17)_4_, (NH_3_^+^)_8_[MPA]-[GMPA](C17)_2_**, and **(NH_3_^+^)_8_[MPA]-[GMPA](C17)_4_** contain ammonium terminated hydrophilic dendrons and stearic acid terminated lipophilic dendrons. In these compounds, the hydrophilic block holds eight ammonium groups at the surface, whereas the lipophilic part contains either two or four stearic acid chains. Besides, the *bis*-MPA/*bis*-GMPA architecture is alternated between both sides and the size of the lipophilic domain varies between two and four chains of stearic acid. The presence of such four chemically different blocks is expected to affect the self-assembly behavior of the Janus dendrimers, their drug loading ability, and their capacity to deliver the drug in target cells. Furthermore, the possibility of exploiting the resulting nanocarriers to rescue the bioactivity of IA and TRIAC, selected in primary screening as potential HCV antivirals, is assessed.

## 2. Materials and Methods

### 2.1. Synthesis and Characterization of the Janus Dendrimers

All reagents, including iopanoic acid (IA) (purity ≥ 95.0%) and 3,5,3′-triiodothyroacetic acid (TRIAC) (purity ≥ 90%) were purchased from Sigma-Aldrich (Saint Louis, MO, USA) and were used without further purification. The solvents were purchased from Scharlab, S.L. (Sentmenat, Spain). MWCO 1000 Dalton regenerated cellulose membrane was purchased from Spectrum^®^ Chemical MFG Corp (New Brunswick, NJ, USA). Tris(benzyltriazolylmethyl)amine (TBTA) catalyst, 4-(dimethylamino)pyridinium 4-toluenesulfonate (DPTS) salt and Merrifield’s peptide resin modified with azide groups were prepared in our laboratory. DMEM (Dulbecco’s modified Eagle’s medium, 4.5 g/L glucose), DPBS (Dulbecco’s phosphate-buffered saline), l-glutamine, 1× non-essential amino acids, and Geneticin (G418) were purchased from Gibco^TM^ (Dublin, Ireland). Penicillin/41 streptomycin (5000 U/mL), amphotericin B (250 mg/mL), and trypsin (trypsin–versene 10×) were obtained from Lonza Group LTD (Basel, Switzerland). Fetal bovine serum was purchased from PAN-Biotech GmbH (Aidenbach, Germany). Bright-Glo^TM^ Luciferase Assay System and CellTiter 96 AQueous One Solution Cell Proliferation Assay were purchased from Promega Corporation (Madison, WS, USA).

^1^H Nuclear Magnetic Resonance (NMR) and ^13^C NMR experiments were performed using an AV-400 (^1^H: 400 MHz, ^13^C: 100 MHz, (Bruker, Billerica, MA, USA) employing deuterated chloroform (CDCl_3_), deuterated dichloromethane (CD_2_Cl_2_), deuterated methanol (CD_3_OD), or deuterated dimethyl sulfoxide ((CD_3_)_2_SO) as solvents. The chemical shifts are given relative to TMS in ppm and the coupling constants in Hz; as internal standard, the solvent residual peak was used. A Microflex system (Bruker, Billerica, MA, USA) was employed to perform the mass spectrometry (MS) studies, using the MALDI-TOF technique with nitrogen laser (337 nm) and dithranol as matrix. Infrared spectra were recorded between 4000 and 600 cm^–1^ on a Vertex 70 spectrophotometer (Bruker, Billerica, MA, USA), which worked in ATR mode. An e2695 Alliance system (Waters Corporation, Milford, MA, USA) was employed to carry out size exclusion chromatography. Two different sets of columns placed in series, Styragel columns HR4 and HR1 (500 and 10^4^ Å of pore size) and two PLgel 5µm MIXED-C Agilent columns (7.5 × 300 mm). A Waters 2424 evaporation light scattering detector was employed with a sample concentration of 1 mg/mL. The solvent was tetrahydrofuran (HPLC grade) with a flow rate of 1 mL/min at 35 °C; PMMA was used as the standard for calibration.

#### 2.1.1. Synthesis of the Dendrons

The synthesis of the intermediates *bis*-MPA lipophilic dendrons with alkyne (**≡-[MPA](C17)_2_** and **≡-[MPA](C17)_4_**) [27] and *bis*-MPA or *bis*-GMPA hydrophilic dendrons with the azide group in the focal point (**N_3_-[MPA](NHBoc)_8_ [28]** and **N_3_-[GMPA](NHBoc)_8_ [26]**) was previously reported by us. The synthesis and chemical characterization of the two novel *bis*-GMPA lipophilic dendrons, **≡-[GMPA](C17)_2_** and **≡-[GMPA](C17)_4_**, and their precursors is gathered in Section S1 of the Supplementary Materials.

#### 2.1.2. Synthesis of the Novel Janus Dendrimers by Cu(I)-Catalyzed Alkyne-Azide Cycloaddition (CuAAC)

CuSO_4_·5H_2_O (0.1 mol), l-ascorbate (0.2 mol) and TBTA (0.1 mol) were dissolved in dry DMF in a Schlenk flask. Three vacuum-argon cycles were carried out to purge the flask form air and the solution was stirred at 45 °C under argon atmosphere for at least 15 min, until it turned yellow in color. The azide (1.0 mol) and alkyne (1.2 mol) dendrons were dissolved in dry DMF at 45 °C in another Schlenk flask and three vacuum-argon cycles were carried out. The previously prepared copper solution was added to the azide-alkyne mixture through a cannula and the reaction mixture was stirred at 45 °C during 24–48 h under argon atmosphere. Then, an excess of Merrifield’s peptide resin modified with azide groups was added to the reaction mixture and after three vacuum-argon cycles, it was stirred at 45 °C during another 24 h in order to remove unreacted alkynes. After that, the reaction mixture was filtered off and the resin was washed with ethyl acetate. 100 mL of brine were added, and the product was extracted twice with ethyl acetate (2 × 70 mL). The organic phases were combined and washed three times with 100 mL each of brine. Then, the organic phase was washed once with a solution of 15 mg of KCN in 100 mL of water) and twice with 100 mL each of brine (2 × 100 mL). The organic phase was dried over anhydrous MgSO_4_ and, after filtration, the solvent was removed by evaporation under reduced pressure. Finally, the crude product was purified by flash chromatography on silica gel using a variable dichloromethane (DCM): methanol (MeOH) ramp ratio (depending on the dendrimer polarity) to give a white or yellow solid.

**(NHBoc)_8_[GMPA]-[MPA](C17)_2_.** N_3_-[GMPA](NHBoc)_8_ (600 mg, 2.35 × 10^−1^ mmol, 1.00 eq.); ≡-[MPA]-(C17)_2_ (199 mg, 2.82 × 10^−1^ mmol, 1.20 equivalent (eq.)); CuSO_4_·5H_2_O (6.9 mg, 2.35 × 10^−2^ mmol, 0.10 eq.); l-ascorbate (9.3 mg, 4.69 × 10^−2^ mmol, 0.20 eq.) and TBTA (12.5 mg, 2.35 × 10^−2^ mmol, 0.10 eq.) into dry DMF (10 mL). The crude product was purified on silica gel (DCM:MeOH ramp from 95:5 to 90:1) to give a yellow solid (646 mg, 84%). ^1^H NMR (400 MHz, CD_2_Cl_2_) δ (ppm): 7.62 (s, 1H, -C_2_*H*_1_N_3_-), 7.10 (bs, -N*H*CO-), 5.46 (bs, -N*H*Boc), 5.22 (s, 2H, -C_2_H_1_N_3_-C*H*_2_-O-), [4.37–4.21] (m, 30H, -CH_2_-C*H*_2_-C_2_H_1_N_3_-, -C*H*_2_-O-), 4.18 (ABq, J = 10.8 Hz, Δν_AB_ = 19.7 Hz, 4H, -C*H*_2_-O-), 4.11 (t, J = 6.4 Hz, 2H, -O-C*H*_2_-CH_2_-), 4.00 (d, J = 5.2 Hz, 8H, -OC(O)-C*H*_2_NHCOO-), 3.95 (d, J = 5.6 Hz, 4H, -OC(O)-C*H*_2_NHCOO-), 3.88 (d, J = 6.0 Hz, 16H, -C*H*_2_NHCOO-C(CH_3_)_3_), 2.24 (t, J = 7.6 Hz, 4H, -OC(O)-C*H*_2_-CH_2_-(CH_2_)_14_-), 1.91 (m, 2H, -C*H*_2_-CH_2_-C_2_H_1_N_3_-), 1.64 (m, 2H, -O-CH_2_-C*H*_2_-), 1.54 (m, 4H, -OC(O)-CH_2_-C*H*_2_-(CH_2_)_14_-), 1.42 (s, 72H, -C(C*H*_3_)_3_), 1.37 (m, 4H, -CH_2_-C*H*_2_-C*H*_2_-CH_2_-), 1.28 (s, 12H, -C-C*H*_3_), 1.26 (m, 65H, -C-C*H*_3_, -(C*H*_2_)_14_-), 1.21 (s, 3H, -C-C*H*_3_), 0.87 (t, J = 8.0 Hz, 6H, -(CH_2_)_16_-C*H*_3_). ^13^C NMR (100 MHz, CD_2_Cl_2_) δ (ppm): [173.6–173.2], 173.1, 170.8, [170.2–170.1], 156.6, 142.9, 124.3, 80.3, [67.1–65.3], 58.9, 50.8, 47.7, [46.8–46.6], 42.9, [41.9–41.8], 34.5, 32.5, 30.6, [30.3–29.7], 28.8, 28.6, 26.6, 25.8, 25.4, 23.3, [18.7–18.1], 14.5. FTIR (ν_max_/cm^−1^, ATR): 3360 (N-H st), 2980-2928-2854 (C-H st), 1747 (C=O st ester), 1713 (C=O st carbamate), 1670 (C=O st amide), 1518 (N-H δ), 1466 (CH_2_, CH_3_ δ), 1367 (C-N st), 1250 (CO-O st), 1157 (N-CO-O st). SEC (ref PMMA): Mw 4056 g·mol^−1^; Ð: 1.02.

**(NHBoc)_8_[GMPA]-[MPA](C17)_4_.** N_3_-[GMPA](NHBoc)_8_ (1000 mg, 3.91 × 10^−1^ mmol, 1.00 eq.); ≡-[*MPA*](C17)_4_ (690 mg, 4.69 × 10^−1^ mmol, 1.20 eq.); CuSO_4_·5H_2_O (11.5 mg, 3.91 × 10^−2^ mmol, 0.10 eq.); (*L*)-ascorbate (15.5 mg, 7.82 × 10^−2^ mmol, 0.20 eq.) and TBTA (20.8 mg, 3.91 × 10^−2^ mmol, 0.10 eq.) into dry DMF (10 mL). The crude product was purified on silica gel (DCM:MeOH ramp from 98:2 to 95:5) to give a yellow solid (1070 mg, 68%). ^1^H NMR (400 MHz, CDCl_3_) δ (ppm): 7.67 (s, 1H, -C_2_*H*_1_N_3_-), 7.19 (bs, -N*H*CO-), 5.25 (bs, -N*H*Boc), 5.22 (s, 2H, -C_2_H_1_N_3_-C*H*_2_-O-), [4.37–4.19] (m, 34H, -CH_2_-C*H*_2_-C_2_H_1_N_3_-, -C*H*_2_-O-), 4.12 (ABq, J = 11.2 Hz, Δν_AB_ = 9.8 Hz, 8H, -C*H*_2_-O-), 4.11 (t, J = 6.0 Hz, 2H, -O-C*H*_2_-CH_2_-), 3.98 (d, J = 5.2 Hz, 8H, -C*H*_2_NHCO_2_-), 3.94 (d, J = 5.2 Hz, 4H, -C*H*_2_NHCO_2_-), 3.88 (d, J = 5.6 Hz, 16H, -C*H*_2_NHCO_2_C(CH_3_)_3_), 2.27 (t, J = 7.6 Hz, 8H, -OC(O)-C*H*_2_-CH_2_-(CH_2_)_14_-), 1.92 (m, 2H, -C*H*_2_-CH_2_-C_2_H_1_N_3_-), 1.63 (m, 2H, -O-CH_2_-C*H*_2_-), 1.56 (m, 8H, -OC(O)-CH_2_-C*H*_2_-(CH_2_)_14_-), 1.44 (s, 72H, -C-(C*H*_3_)_3_), 1.37 (m, 4H, -CH_2_-C*H*_2_-C*H*_2_-CH_2_-), 1.27 (s, 12H, -C-C*H*_3_), [1.26–1.16] (m, 130H, -C-C*H*_3_, -(C*H*_2_)_14_-), 0.86 (t, J = 6.4 Hz, 12H, -(CH_2_)_16_-C*H*_3_). ^13^C NMR (100 MHz, CDCl_3_) δ (ppm): [173.1–169.5], 156.0, 141.9, 124.1, 80.0, [66.7–64.9], 58.4, 50.1, [46.6–46.0], 42.3, [41.3–41.2], 34.0, 31.9, 30.1, [29.7–29.1], 28.3, 26.0, 25.2, 24.8, 22.7, [18.3–17.7], 14.1. FTIR (ν_max_/cm^−1^, ATR): 3362 (N-H st), 2976-2918-2851 (C-H st), 1744 (C=O st ester), 1717 (C=O st carbamate), 1663 (C=O st amide), 1526 (N-H δ), 1470 (CH_2_, CH_3_ δ), 1367 (C-N st), 1252 (CO-O st), 1159 (N-CO-O st). SEC (*ref PMMA*): Mw 4952 g·mol^−1^; Ð: 1.02.

**(NHBoc)_8_[MPA]-[GMPA](C17)_2_.** N_3_-[MPA](NHBoc)_8_ (900 mg, 4.07 × 10^−1^ mmol, 1.00 eq.); ≡-[GMPA](C17)_2_ (400 mg, 4.88 × 10^−1^ mmol, 1.20 eq.); CuSO_4_·5H_2_O (12.0 mg, 4.07 × 10^−2^ mmol, 0.10 eq.); *(L)*-ascorbate (16.1 mg, 8.14 × 10^−2^ mmol, 0.20 eq.) and TBTA (27.6 mg, 4.07 × 10^−2^ mmol, 0.10 eq.) into dry DMF (10 mL). The crude product was purified through silica gel column chromatography (DCM:MeOH ramp from 100:0 to 96:4) to obtain a white powder (334 mg, 27%). ^1^H NMR (400 MHz, CDCl_3_) δ (ppm): 7.74 (s, 1H, -C_2_*H*_1_N_3_-), 6.43 (bs, -N*H*CO-), 5.38 (bs, -N*H*Boc), 5.25 (s, 2H, -C_2_H_1_N_3_-C*H*_2_-O-), 4.39 (t, J = 6.0 Hz, 2H, -CH_2_-C*H*_2_-C_2_H_1_N_3_-), 4.26 (m, 32H, -C*H*_2_-O-), 4.20 (t, J = 6.0 Hz, 2H, -O-C*H*_2_-CH_2_-), 3.88 (s, 20H, -C*H*_2_NHCO_2_C(CH_3_)_3_, -C*H*_2_NHCO_2_-), 2.24 (t, J = 8.0 Hz, 4H, -OC(O)-C*H*_2_-CH_2_-(CH_2_)_14_-), 1.80 (t, J = 5.0 Hz, 2H, -C*H*_2_-CH_2_-C_2_H_1_N_3_-), 1.64 (m, 6H, -O-CH_2_-C*H*_2_-), -OC(O)-CH_2_-C*H*_2_-(CH_2_)_14_-), 1.44 (s, 72H, -C-(C*H*_3_)_3_), 1.28 (s, 12H, -C-C*H*_3_), 1.25 (s, 72H, -C-C*H*_3_, -CH_2_-C*H*_2_-C*H*_2_-CH_2_-, -(C*H*_2_)_14_-), 0.88 (t, J = 6.8 Hz, 6H, -(CH_2_)_16_-C*H*_3_). ^13^C NMR (100 MHz, CDCl_3_) δ (ppm)**:** 173.9, 172.4, 172.2, 172.0, 171.7, 170.2, 169.7, 156.0, 80.1, 65.8, 65.5, 46.5, 42.4, 41.2, 36.4, 32.0, 29.8, 29.7, 28.5, 25.7, 22.8, 18.1, 17.7, 14.2. FTIR (ν_max_/cm^−1^, ATR): 3370 (N-H st), 2922 and 2852 (C-H st), 1742 (C=O st ester), 1718 (C=O st carbamate), 1522 (N-H δ), 1470 (CH2, CH3 δ), 1367 (C-N st), 1157 (N-CO-O st). SEC (ref PMMA): Mw 6346 g·mol^−1^; Ð 1.05.

**(NHBoc)_8_[MPA]-[GMPA](C17)_4_**. N_3_-[MPA](NHBoc)_8_ (334 mg, 1.51 × 10^−1^ mmol, 1.00 eq.); ≡-[GMPA](C17)_4_ (328 mg, 1.81 × 10^−1^ mmol, 1.20 eq.); CuSO_4_·5H_2_O (3.8 mg, 1.51 × 10^−2^ mmol, 0.10 eq.); (*L*)-ascorbate (6.0 mg, 3.02 × 10^−2^ mmol, 0.20 eq.) and TBTA (8.0 mg, 1.51 × 10^−2^ mmol, 0.10 eq.) into dry DMF (10 mL). The crude product was purified on silica gel (DCM:MeOH ramp from 100:0 to 98:2) to give a yellow solid (380 mg, 63%). ^1^H NMR (400 MHz, CDCl_3_) δ (ppm): 7.76 (s, 1H, -C_2_*H*_1_N_3_-), 6.62 (bs, -N*H*CO-), 5.39 (bs, -N*H*Boc), 5.25 (s, 2H, -C_2_H_1_N_3_-C*H*_2_-O-), 4.39 (t, J = 7.4 Hz, 2H, -CH_2_-C*H*_2_-C_2_H_1_N_3_-), [4.35–4.18] (m, 40H, -C*H*_2_-O-), 4.13 (t, J = 6.8 Hz, 3H, -O-C*H*_2_-CH_2_-), 4.03 (d, J = 6.2 Hz, 8H, -C*H*_2_NHCO_2_-), 3.94 (d, J = 5.6 Hz, 4H, -C*H*_2_NHCO_2_-), 3.87 (d, J = 6.2 Hz, 16H, -C*H*_2_NHCO_2_C(CH_3_)_3_), 2.24 (t, J = 7.7 Hz, 8H, -OC(O)-C*H*_2_-CH_2_-(CH_2_)_14_-), 1.94 (m, 2H, -C*H*_2_-CH_2_-C_2_H_1_N_3_-), 1.62 (m, 10H, -O-CH_2_-C*H*_2_-, -OC(O)-CH_2_-C*H*_2_-(CH_2_)_14_-), 1.44 (s, 72H, -C-(C*H*_3_)_3_), 1.28 (s, 12H, -C-C*H*_3_), 1.25 (s, 134H, -C-C*H*_3_, -CH_2_-C*H*_2_-C*H*_2_-CH_2_-, -(C*H*_2_)_14_-), 0.87 (t, J = 7.1 Hz, 12H, -(CH_2_)_16_-C*H*_3_). ^13^C NMR (100 MHz, CDCl_3_) δ (ppm): [174.4–169.3], 155.9, 80.0, [65.9–65.4], 46.7, 46.4, 45.9, 42.2, 41.4, 36.2, 31.9, [29.7–29.4], 28.3, 25.7, 22.7, [18.1–17.6], 14.1. FTIR (ν_max_/cm^−1^, ATR): 3355 (N-H st), 2960-2918-2850 (C-H st), 1735 (C=O st ester), 1718 (C=O st carbamate), 1656 (C=O st amide), 1521 (N-H δ), 1467 (CH_2_, CH_3_ δ), 1365 (C-N st), 1265-1220 (CO-O st), 1157 (N-CO-O st). SEC (ref PMMA): Mw 4757 g·mol^−1^; Ð: 1.02.

#### 2.1.3. Deprotection of Amine Groups

Deprotection method A. The *t*-Boc protected amino-terminated dendrimer (1 mol) was dissolved into ethyl acetate (5 mL) and a saturated solution of HCl in ethyl acetate (10 mL) was carefully added to it. The reaction mixture was stirred at room temperature. After 1 h, a white precipitate appeared, and 35 mL of ethyl acetate was then added. The mixture was stirred for 30 min, after which the hydrochloric acid was removed under vacuum. The white precipitate was separated by centrifugation and was washed twice with ethyl acetate. This method was employed to obtain the final products **(NH_3_^+^)_8_[GMPA]-[MPA](C17)_2_, (NH_3_^+^)_8_[GMPA]-[MPA](C17)_4_**, and **(NH_3_^+^)_8_[MPA]-[GMPA](C17)_2_.**

Deprotection method B. The aforementioned deprotection method resulted into partial structural cleavage in the case of **(NH_3_^+^)_8_[MPA]-[GMPA](C17)_4_**. Hence, an alternative deprotection method was employed in this case [29]. It consisted of the dissolution of the *t*-Boc-protected dendrimer in a mixture of chloroform and trifluoroacetic acid (1:1 in volume). The reaction proceeded at room temperature for 1 to 5 h depending on the dendron generation after which the solvent and the excess of trifluoroacetic acid were removed under vacuum. The product was then dissolved in dichloromethane or methanol and precipitated in cold ether. After decanting, the remaining solvent traces were removed under vacuum.

**(NH_3_^+^)_8_[GMPA]-[MPA](C17)_2_.** Deprotection method A. (NHBoc)_8_[GMPA]-[MPA](C17)_2_ (424 mg, 1.30 × 10^−1^ mmol, 1.00 eq.). The product was obtained as a white solid (313 mg, quantitative yield). ^1^H NMR (400 MHz, CD_3_OD) δ (ppm): 8.08 (s, 1H, -C_2_*H*_1_N_3_-), 5.26 (s, 2H, -C_2_H_1_N_3_-C*H*_2_-O-), 4.45 (m, 18H, -CH_2_-C*H*_2_-C_2_H_1_N_3_-, -C*H*_2_-O-), 4.32 (m, 12H, -C*H*_2_-O-), 4.21 (ABq, J = 11.2 Hz, ΔνAB = 27.7 Hz, 4H, -C*H*_2_-O-), 4.13 (t, J = 6.4 Hz, 2H, -O-C*H*_2_-CH_2_-), 4.01 (s, 8H, -C*H*_2_NHCO_2_-), 3.95 (s, 16H, -C*H*_2_NH_3_^+^), 3.97 (s, 4H, -C*H*_2_NHCO_2_-), 2.26 (t, 4H, J = 7.2 Hz, -OC(O)-C*H*_2_-CH_2_-(CH_2_)_14_-), 1.94 (m, 2H, -C*H*_2_-CH_2_-C_2_H_1_N_3_-), 1.67 (m, 2H, -O-CH_2_-C*H*_2_-), 1.55 (m, 4H, -OC(O)-CH_2_-C*H*_2_-(CH_2_)_14_-), 1.44 (m, 4H, -CH_2_-C*H*_2_-C*H*_2_-CH_2_-), 1.38 (s, 12H, -C-C*H*_3_), 1.32 (s, 6H, -C-C*H*_3_), 1.29 (m, 56H, -(C*H*_2_)_14_-), 1.27 (s, 3H, -C-C*H*_3_), 1.24 (s, 3H, -C-C*H*_3_), 0.90 (t, J = 6.8 Hz, 6H, -(CH_2_)_16_-C*H*_3_). ^13^C NMR (100 MHz, CD_3_OD) δ (ppm): [175.2–174.6], [174.1–174.0], [171.1–170.8], 168.3, 143.5, 126.2, 68.5, 67.8, 67.2, [66.4–66.2], 58.9, 51.4, [47.6–47.4], 42.2, 41.2, 34.8, 33.1, 31.1, [30.8–30.1], 29.4, 27.0, 26.4, 26.0, 23.7, [18.2–17.8], 14.5. MS (MALDI^+^) *m/z (%):* found 2482.9(60), calculated for [C_113_H_191_N_17_O_42_,Na]^+^ *2481.3*. FTIR (ν_max_/cm^−1^, ATR): 3600-2600 (bs N-H^+^ st), 2920-2852 (C-H st), 1745 (C=O st ester), 1653 (C=O st amide and N-H^+^ δ), 1537 (N-H δ), 1468 (CH_2_, CH_3_ δ), 1229 (CO-O st).

**(NH_3_^+^)_8_[GMPA]-[MPA](C17)_4_.** Deprotection method A. (NHBoc)_8_[GMPA]-[MPA](C17)_4_ (290 mg, 7.20 × 10^−2^ mmol, 1.00 eq.). The product was obtained as a white solid (253 mg, quantitative yield). ^1^H NMR (400 MHz, CD_3_OD) δ (ppm): 8.07 (s, 1H, -C_2_*H*_1_N_3_-), 7.33 (bs, -N*H*CO-), 5.26 (s, 2H, -C_2_H_1_N_3_-C*H*_2_-O-), 4.45 (m, 18H, -CH_2_-C*H*_2_-C_2_H_1_N_3_-, -C*H*_2_-O-), 4.32 (m, 12H, -C*H*_2_-O-), 4.27 (ABq, 4H, -C*H*_2_-O-), 4.13 (m, 10H, -O-C*H*_2_-CH_2_-, -C*H*_2_-O-), 4.00 (m, 8H, -C*H*_2_NHCO_2_-), 3.97 (m, 4H, -C*H*_2_NHCO_2_-), 3.94 (s, 16H, -C*H*_2_NH_3_^+^), 2.33 (t, J = 7.2Hz, 8H, -OC(O)-C*H*_2_-CH_2_-(CH_2_)_14_-), 1.95 (m, 2H, -C*H*_2_-CH_2_-C_2_H_1_N_3_-), 1.67 (m, 2H, -O-CH_2_-C*H*_2_-), 1.59 (m, 8H, -OC(O)-CH_2_-C*H*_2_-(CH_2_)_14_-), 1.43 (m, 4H, -CH_2_-C*H*_2_-C*H*_2_-CH_2_-), 1.38 (s, 12H, -C-C*H*_3_), [1.33-1.28] (m, 124H, -C-C*H*_3_, -(C*H*_2_)_14_-), 1.20 (s, 6H, -C-C*H*_3_), 0.90 (t, J = 6.8Hz, 12H, -(CH_2_)_16_-C*H*_3_). ^13^C NMR (100 MHz, CD_3_OD) δ (ppm): [175.3-170.8], 168.3, 143.5, 126.3, [68.6-66.3], 59.1, 51.4, [47.9-47.4], 42.2, 41.2, 34.9, 33.1, 31.2, [30.9-30.2], 29.4, 27.1, 26.4, 26.1, 23.8, [18.3-17.8], 14.5. MS (MALDI^+^) *m/z (%):* found *3248.6(100)* calculated for [C_159_H_275_N_17_O_50_,Na]^+^ *3246.0.* FTIR (ν_max_/cm^−1^, ATR): 3000–2600 (bs N-H^+^ st), 2959-2918-2851 (C-H st), 1738 (C=O st ester), 1653 (C=O st amide and N-H^+^ δ), 1539 (N-H δ) 1470 (CH_2_, CH_3_ δ), 1217 (CO-O st), 1132 (O-C-C st).

**(NH_3_^+^)_8_[MPA]-[GMPA](C17)_2_.** Deprotection method A. (NHBoc)_8_[MPA]-[GMPA](C17)_2_ (280 mg, 9.23 × 10^−2^ mmol, 1.00 eq.). The product was obtained as a white solid (220 mg, quantitative yield). ^1^H NMR (400 MHz, CD_3_OD) δ (ppm): 8.10 (s, 1H, -C_2_*H*_1_N_3_-), 5.27 (s, 2H, -C_2_H_1_N_3_-C*H*_2_-O-), [4.50–4.39] (m, 18H, -CH_2_-C*H*_2_-C_2_H_1_N_3_-, -C*H*_2_-O-), [4.40-4.22] (m, 16H, -C*H*_2_-O-), 4.15 (t, J = 6.0 Hz, 2H, -O-C*H*_2_-CH_2_-), 3.96 (s, 16H, -C*H*_2_NH_3_^+^), 3.88 (s, 4H, -C*H*_2_NHCO_2_-), 2.26 (t, J = 7.0 Hz, 4H, -OC(O)-C*H*_2_-CH_2_-(CH_2_)_14_-), 1.95 (m, 2H, -C*H*_2_-CH_2_-C_2_H_1_N_3_-), 1.69 (m, 2H, -O-CH_2_-C*H*_2_-), 1.62 (m, 4H, -OC(O)-CH_2_-C*H*_2_-(CH_2_)_14_-), 1.45 (m, 4H, -CH_2_-C*H*_2_-C*H*_2_-CH_2_-), 1.33 (s, 12H, -C-C*H*_3_), 1.29 (m, 65H, -C-C*H*_3_, -(C*H*_2_)_14_-), 1.25 (s, 3H, -C-C*H*_3_), 0.90 (t, J = 6.6 Hz, 6H, -(CH_2_)_16_-C*H*_3_). ^13^C NMR (100 MHz, CD_3_OD) δ (ppm): 176.8, 173.9, 173.8, 173.4, 173.2, 170.8, 168.4, 143.6, 126.2, 67.6, 66.8, 59.1, 51.3, 47.6, 41.9, 41.2, 36.8, 33.1, 31.1, [30.9-30.1], 27.0, 26.4, 23.7, 18.2, 14.5. MS (MALDI^+^) *m/z (%):* found *2231.9 (100),* calculated for [C_105_H_179_N_13_O_38_,H]^+^ *2232.2* FTIR (ν_max_/cm^−1^, ATR): 3674-3202 (N-H st), 3202-2600 (N-H^+^ st), 2962-2920-2851 (C-H st), 1734 (C=O st ester), 1647 (C=O amide st), 1472 (CH_2_, CH_3_ δ), 1217 (C-O st), 1128 (O-C-C st).

**(NH_3_^+^)_8_[MPA]-[GMPA](C17)_4_.** Deprotection method B. (NHBoc)_8_[MPA]-[GMPA](C17)_4_ (360 mg, 8.9 × 10^−2^ mmol, 1.00 eq.). The product was obtained as a white solid (290 mg, quantitative yield). ^1^H NMR (400 MHz, CD_3_OD) δ (ppm): 8.09 (s, 1H, -C_2_*H*_1_N_3_-), 5.27 (s, 2H, -C_2_H_1_N_3_-C*H*_2_-O-), 4.43 (m, 18H, -CH_2_-C*H*_2_-C_2_H_1_N_3_-, -C*H*_2_-O-), 4.30 (m, 24H, -C*H*_2_-O-), 4.14 (t, J = 6.5 Hz, 2H, -O-C*H*_2_-CH_2_-), 3.95 (s, 8H, -C*H*_2_NHCO_2_-), 3.94 (s, 16H, -C*H*_2_NH_3_^+^), 3.90 (s, 4H, -C*H*_2_NHCO_2_-), 2.26 (t, J = 7.6 Hz, 8H, -OC(O)-C*H*_2_-CH_2_-(CH_2_)_14_-), 1.95 (m, 2H, -C*H*_2_-CH_2_-C_2_H_1_N_3_-), 1.68 (m, 2H, -O-CH_2_-C*H*_2_-), 1.62 (m, 8H, -OC(O)-CH_2_-C*H*_2_-(CH_2_)_14_-), 1.45 (m, 4H, -CH_2_-C*H*_2_-C*H*_2_-CH_2_-), 1.29 (m, 139H, -C-C*H*_3_, -(C*H*_2_)_14_-), 1.26 (s, 3H, -C-C*H*_3_), 0.90 (t, J = 6.9 Hz, 12H, -(CH_2_)_16_-C*H*_3_). ^13^C NMR (100 MHz, CD_3_OD) δ (ppm): 176.8, 173.3, 173.2, 171.0, 170.7, 168.5, 163.0, 162.7, 119.5, 67.5, 66.7, 51.3, 48.1, 47.7, 47.3, 42.1, 41.0, 36.8, 33.1, [30.8-30.4], 26.9, 26.5, 23.8, 18.0, 14.5. MS (MALDI^+^) *m/z (%):* found *3246.8 (100)*, calculated for [C_159_H_275_N_17_O_50_,Na]^+^ *3246.0*. FTIR (ν_max_/cm^−1^, ATR): 3623-2352 (bs N-H^+^ st), 2918-2850 (C-H st), 1745 (C=O st ester), 1674 (C=O st amide and N-H^+^ δ), 1537 (N-H δ), 1469 (CH_2_, CH_3_ δ), 1199 (CO-O st), 1130 (O-C-C st).

### 2.2. Formation and Characterization of the Dendrimer Aggregates

#### 2.2.1. Critical Aggregation Concentration (CAC) Determination

The critical aggregation concentration (CAC) of the amphiphilic Janus dendrimers was measured using the method based on nile red fluorescence [30]. The solutions of the Janus dendrimers were prepared at different concentrations in the range from 1 × 10^–5^ to 1 mg/mL. 10 µL of a solution of nile red in ethanol at a concentration of 2.5 × 10^–1^ mM was added to each dendrimer solutions. The resulting solutions were stirred for 1 h at room temperature in the dark using an orbital shaker. The emission spectrum was recorded (λ_max_ = 635 nm and λ_exc_ = 550 nm), and the CAC was determined from the plots representing the fluorescence emission intensity of nile red as a function of the dendrimer concentration. The change of the curve slope corresponds to the beginning of lipophilic domain formation. The fluorescence emission spectra were recorded on a -Ls 55 system (PerkinElmer, Waltham, MA, USA).

#### 2.2.2. Formation and Morphology of the Dendrimer Aggregates

The oil-in-water method [28] was employed to prepare the dendrimer aggregates of each amphiphilic Janus dendrimer using dichloromethane as organic solvent. Each dendrimer was dissolved at a concentration of 1 mg/mL in dichloromethane and milliQ water was added in the appropriate volume to obtain a final concentration of 1 mg/mL. The organic solvent was completely evaporated by stirring the mixtures at room temperature with an orbital shaker.

The hydrodynamic diameters of the dendritic nanocarriers were measured with a Nano ZS (Malvern Instruments, Malvern, UK) with a 633 nm wavelength laser and a detection angle of 173°. The samples were dissolved in distilled water at the concentration of 1 mg/mL. Three scans of 5 min each were carried out for each sample. Average hydrodynamic diameters, D_H_, were obtained after applying number data treatment.

Transmission electron microscopy (TEM) images were recorded on a TECNAI T20 system (FEI^TM^, Hillsboro, OR, USA) with a beam power of 200 kV. A droplet (10 μL) of the freshly prepared sample at 1 mg/mL was deposited on a Formvar (10 nm)/Carbon Film (1 nm) coated 400 mesh coppered grid (ANAME Intrumentación Cientifica, Quijorna, Spain) and 1% aqueous uranyl acetate solution was used as negative stain.

### 2.3. Formation and Characterization of Drug-Loaded Dendrimer Aggregates

#### 2.3.1. Isothermal Titration Calorimetry (ITC)

The interaction between the different drugs and dendritic aggregates was characterized using an Auto-iTC200 microcalorimeter (Malvern Instruments, Malvern, UK). Dendritic aggregate in the calorimetric cell at 20–80 µM was titrated with drug at 200–1600 at 50 µM. All solutions were dissolved in PBS/milliQ H_2_O (1:1 in volume) with up to 2% of DMSO and degassed at 25 °C for 2 min before each assay. A sequence of 2 µL-injections of titrant solution every 150 s was programmed and the stirring speed was set to 750 rpm. The association constant (K_a_) and the enthalpy change (ΔH) were estimated through non-linear regression of the experimental data employing a single ligand binding site model (1:1 dendrimer aggregates:drug stoichiometry) implemented in Origin (OriginLab, Northampton, MA, USA) [31].

#### 2.3.2. Drug Loading

The solvent diffusion technique was employed to load the compounds IA and TRIAC within the dendritic aggregates previously formed. Firstly, drugs were dissolved into DMSO at the high concentration of 50 mg/mL (87.58 mM IA and 80.39 mM TRIAC, respectively). An accurate volume of each drug-containing solution was added to the previously prepared aggregates to reach the ratio of 5 mol of drug per mol of dendrimer, ensuring also that DMSO volume in the mixture did not exceed 2.5% (*v*/*v*). The mixtures were stirred at 4 °C for 16 h to allow drugs to enter within the nanocarriers. DMSO was removed by dialysis against distilled water in sink conditions at 4 °C using a MWCO 1000 Dalton membrane and replacing dialysis medium three times up to 48 h. The non-entrapped compounds were removed by filtration through 0.22 μm syringe filter of regenerated cellulose to obtain the drug loaded nanocarriers.

The concentration of loaded drug was directly determined by UV-Vis spectroscopy using a CaryBio 100 UV-Vis spectrophotometer (Agilent Technologies, Santa Clara, CA, USA). Absorbance measurements were carried out at λ_IA_ = 317 nm and λ_TRIAC_ = 300 nm by adding 75% (*v*/*v*) of spectrophotometric grade DMSO (Sigma-Aldrich, Saint Louis, MO, USA) to dissociate the aggregates and allow the release of the drug. In each case, the absorbance was compared with a calibration curve in the range between 15 and 125 μg/mL of the corresponding drug (Figure S8). The loading process was repeated at least three times, and the average drug loading content (DLC) was determined as the molar ratio of loaded drug per Janus dendrimer. The morphology of the drug-loaded nanocarriers was studied by TEM.

### 2.4. Antiviral Studies

#### 2.4.1. Cells and Replicon System

The highly permissive cell clone Huh 7-Lunet, as well as Huh 7 cells containing subgenomic HCV replicons I389luc-ubi-neo/NS3-3′/5.1 (Huh 5-2) (a kind gift from Dr. V. Lohmann and Dr. R. Bartenschlager) has been previously described [32,33,34,35]. Briefly, this system allows for quantifying the amount of RNA transcribed and translated using luciferase as reporter. Virus replication is proportional to the luminescence detected. Cells culture media were DMEM supplemented with 10% heat-inactivated fetal bovine serum, 1x non-essential amino acids, 100 IU/mL penicillin, 100 µg/mL streptomycin and 250 µg/mL geneticin (G418).

#### 2.4.2. Antiviral Assay with Huh 5-2 Cells

Antiviral assays for assessing the efficacy of the drug-dendrimer systems were performed as described in the literature [32,33,34,35,36]. Briefly, 7 × 10^3^ of Huh 5-2 cells per well were seed in a tissue culture-treated white 96-well view plate (Techno Plastic Products AG, Trasadingen, Switzerland). In this case, to avoid interactions with the luminescence detection reagent, DMEN without phenol red was used. The medium was removed after incubation for 24 h at 37 °C and drug-dendrimer nanocarriers solutions (two-fold serial dilutions) in complete DMEM (without G418) were added to a total volume of 100 µL.

The final drug concentrations up to 160 µM for IA and up to 67 µM for TRIAC were tested, the maximum assayed concentration depended on the loading efficiency. Specifically, 160, 80, and 99 µM of IA loaded into **(NH_3_^+^)_8_[GMPA]-[MPA](C17)_2_, (NH_3_^+^)_8_[GMPA]-[MPA](C17)_4_** and **(NH_3_^+^)_8_[MPA]-[GMPA](C17)_2_**, respectively, were assayed. For TRIAC, 63, 67, and 61 µM were tested within the dendrimer aggregates. After 3 days of incubation at 37 °C, luciferase activity was determined using the Bright-Glo^TM^ Luciferase Assay System (30 µL per well) (Promega Corporation, Madison, WS, USA). The luciferase signal was measured using a Synergy HT 50 Multimode Reader (BioTek Instruments, Winooski, VT, USA). Luminescence signal levels obtained in each assay were normalized using internal patterns previously determined in Huh 5-2 cells. The 40% effective concentration (EC40) was defined as the concentration of compound that reduced the luciferase signal by 40%.

### 2.5. Cell Viability Assay

The cellular viability of all the dendrimer/drug aggregates and the empty dendrimer aggregates was assessed in human cancer cell lines (a kind gift from Dr. V. Lohmann and Dr. R. Bartenschlager). Briefly, Huh 5-2 cells were seeded at a density of 7 × 10^3^ cells per well of a 96-well plate in complete DMEM with the appropriate concentration of G418. Serial dilutions of the corresponding aggregate in complete DMEM (without G418) were added 24 h after seeding. The cells were allowed to proliferate for 3 days at 37 °C. Then, the cell culture medium was removed, and cell number was determined by CellTiter 96^®^ AQueous One Solution Cell Proliferation Assay (Promega Corporation, Madison, WS, USA). The 50% cytostatic concentration (CC50) was defined as the concentration in which 50% of initial cell viability was reached. All experiments on Huh 5-2 cells were carried out in triplicate and each experiment was repeated on three different days.

### 2.6. Cytometry Assay

An ImageStream X Amnis System Cytometer (EMD Millipore, Seattle, WA, USA) was used to determine rhodamine internalization levels and DNA staining with propidium iodide. The procedure employed to label the dendrimer aggregates with RhB(C17)_2_ is gathered in the Supplementary Materials. Image X Amnis is an image multispectral cytometer which is able to take a huge number of digital images for each sample. We can obtain an image for each cell, so this is a statistically powerful device. The IDEAS^®^ software (EMD Millipore, Seattle, WA, USA) was used to analyze cytometry images. Cytometry assays were performed in a similar way to antiviral and cell viability tests. After being seeded, cells were incubated with serial dilutions of **(NH_3_^+^)_8_[GMPA]-[MPA](C17)_4_** and **(NH_3_^+^)_8_[GMPA]-[MPA](C17)_4_/RhB(C17)_2_**, up to 3.7 µM of dendrimer aggregates (corresponding to 1.5 µM of RhB(C_17_)_2_) for 3 days at 37 °C. Moreover, a control only treated with DMEM was introduced. The cells were collected in a volume of 200 µL and were washed three times with PBS by centrifugation at 1500 rpm to remove trypsin. Finally, the samples were analyzed in the ImageStream X Amnis Cytometer.

Rhodamine was excited using a laser at 488 nm and emitted fluorescence was detected at 529 nm in the appropriate channel. The laser power was set at 10 mW to detect rhodamine fluorescence inside the cells.

DNA was stained with propidium iodide dye in order to analyze cell cycle in treated cells. Propidium iodide binds stoichiometrically to DNA, allowing us to detect cells in different stages of their cell cycle and determine the if cell replication machinery is working properly or if the DNA has been damaged. For this, 30 µL of propidium iodide solution (1 mg/mL) was added to each sample. The samples were shaken in a vortex and incubated in darkness for 3 min before introducing in the cytometer. Propidium iodide was excited using a laser that emits at 488 nm and emitted fluorescence was detected at 660 nm in another channel to avoid emission fluorescence overlap rhodamine. The laser power was set at 10 mW to detect propidium iodide. The right morphology and cells complexity were selected using brightfield and sidescatter channels (SSC). When a homogeneous cell population was obtained, IDEAS^®^ software was used to determine rhodamine internalization levels and to analyze the cell cycle.

## 3. Results and Discussion

### 3.1. Synthesis and Chemical Characterization of the Janus Dendrimers

The *bis*-MPA dendrons of 1st and 2nd generation, **≡-[MPA](C17)_2_** and **≡-[MPA](C17)_4_**, bearing lipophilic C_17_H_35_ chains and an alkyne group at the focal point [27], and the hydrophilic *bis*-MPA derived amino-terminated dendron of 3rd generation **N_3_-[MPA](NHBoc)_8_** with an azide group at the focal point [28], were synthesized following our previously described procedures [27,28]. Briefly, *bis*-MPA dendrons were prepared by the repetition of esterification reactions, carried out with *N*,*N*′-dicyclohexylcarbodiimide (DCC) and DPTS, followed by hydrolysis of the ketal group under mild conditions to deprotect the hydroxyl groups. Functionalization at their periphery by esterification with an excess of 1.50 eq. of stearic acid per hydroxyl group, together with 4-dimethylaminopyridine (DMAP) and DCC, rendered the lipophilic units [27]. Peripheral esterification of the hydroxyl groups with *t*-Boc-glycine yielded the *bis*-MPA hydrophilic unit [28]. The *t*-Boc-amino-terminated *bis*-GMPA dendron of third generation **N_3_-[GMPA](NHBoc)_8_** was synthesized from 6-azidohexyl *bis*(hydroxymethyl)propionate by the repetition of amidation coupling reactions with *t*-Boc-glycine moieties, carried out in the presence of DCC, 1-hydroxybenzotrizole (HOBt), and DMAP, followed by the deprotection of the amino groups in acidic conditions, as we described previously [26].

**Scheme 1:**
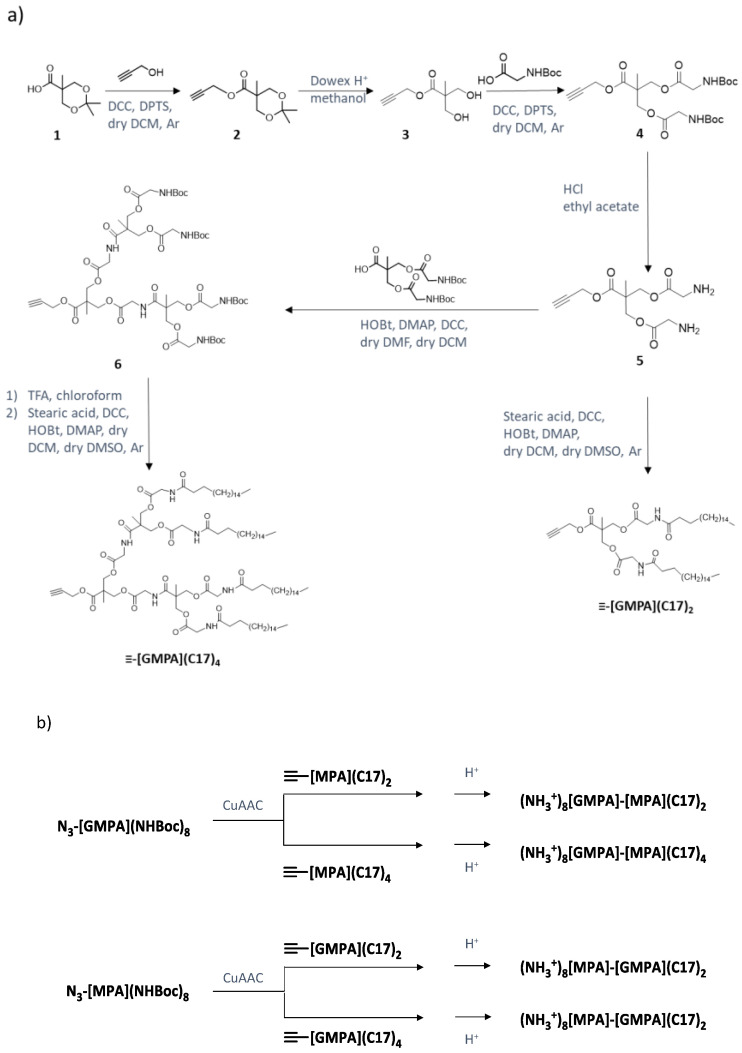
(**a**) Scheme of the synthesis of the two novel lipophilic *bis*-GMPA dendrons with terminal stearic acid chains and alkyne group in the focal point: **≡-[GMPA](C17)_2_** and **≡-[GMPA]-(C17)_4_**. (**b**) Synthetic route for the synthesis of the final Janus dendrimers.

The two novel 1st and 2nd generation dendrons based on *bis*-GMPA with lipophilic C_17_H_35_ chains, **≡-[GMPA](C17)_2_** and **≡-[GMPA](C17)_4_**, were synthesized as outlined in Scheme 1a. Starting from propargyl *bis*(hydroxymethyl)propionate [27], **3**, the repetition of amidation coupling reactions with *t*-Boc-glycine moieties and subsequent deprotection of the amino groups yielded the alkyne-functionalized dendrons **≡-[GMPA](NH_3_^+^)_2_** and **≡-[GMPA](NH_3_^+^)_4_**, which were finally functionalized by amidation at their periphery with 2 and 4 stearic acid chains, respectively, with similar reaction conditions as described above (synthetic details are described in Section S1 of the Supplementary Materials).

The alkyne and *t*-Boc-protected azide dendrons were linked by copper(I)-catalyzed azide-alkyne cycloaddition (CuAAC) to obtain the four new amphiphilic Janus dendrimers **(NHBoc)_8_[GMPA]-[MPA](C17)_2_, (NHBoc)_8_[GMPA]-[MPA](C17)_4_**, **(NHBoc)_8_[MPA]-[GMPA](C17)_2_**, and **(NHBoc)_8_[MPA]-[GMPA](C17)_4_** (Scheme 1b). Copper(I) was obtained in situ by reduction of copper(II) sulfate by (*L*)-sodium ascorbate. Tris[(1-benzyl-1*H*-1,2,3-triazol-4-yl)methyl]amine (TBTA) was added to the reaction mixture to increase the stability of copper(I), since this catalytic strategy has proven to be advantageous for the design of dendrimers [37]. Finally, the *t*-Boc protecting groups were removed under acidic conditions to obtain the corresponding ammonium salts in quantitative yields. **(NHBoc)_8_[GMPA]-[MPA](C17)_2_, (NHBoc)_8_[GMPA]-[MPA](C17)_4_**, and **(NHBoc)_8_[MPA]-[GMPA](C17)_2_** were deprotected using HCl to cleave the *t*-Boc groups (deprotection method A in Section 2.1.3). However, this procedure resulted in structural ruptures of **(NHBoc)_8_[MPA]-[GMPA](C17)_2_** and trifluoroacetic acid was employed instead (deprotection method B in Section 2.1.3).

**Figure 2:**
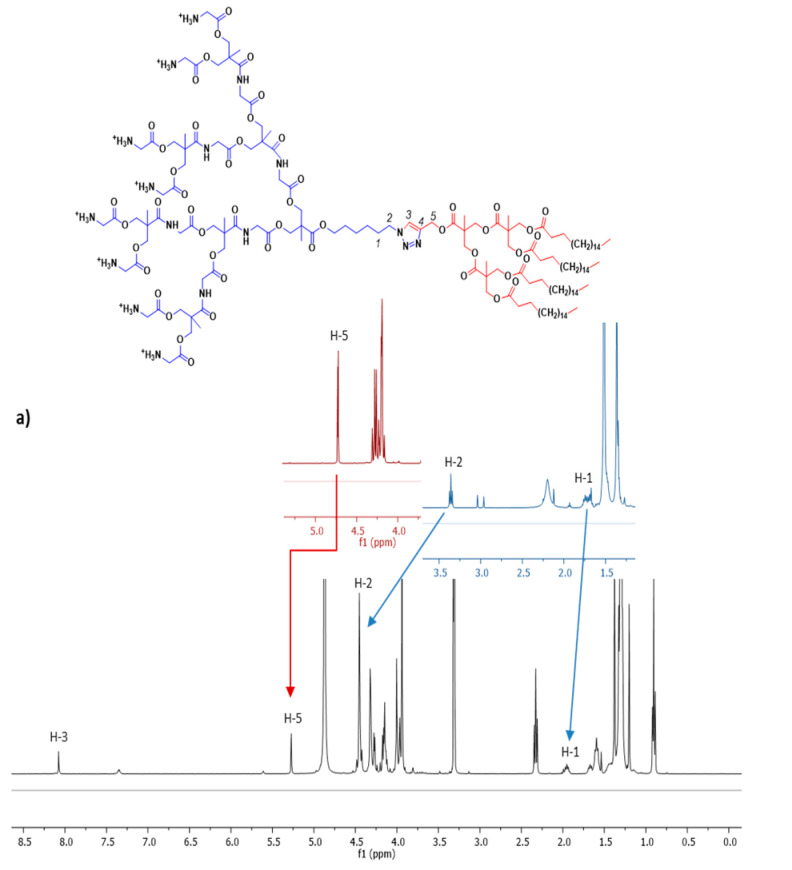

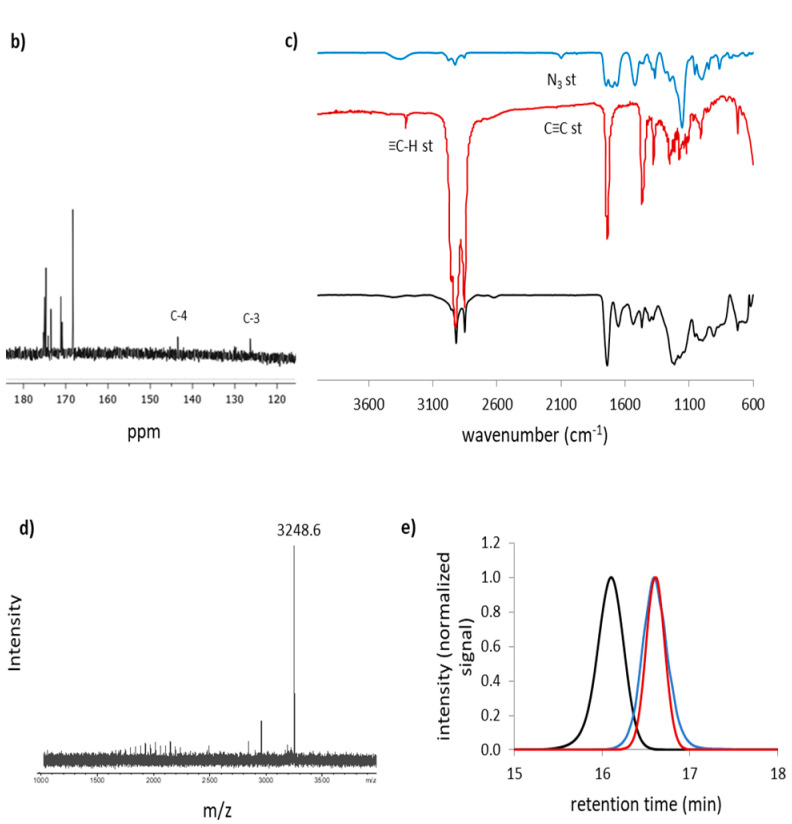
(**a**) ^1^H NMR spectrum of **(NH_3_^+^)_8_[GMPA]-[MPA](C17)_4_** dendrimer from 8.5 to 0.0 ppm in CD_3_OD recorded at 400 MHz. Main chemical shifts of key signals with respect to those in the starting dendrons are also indicated (colored fragments of each spectrum recorded in CDCl_3_ at 400 MHz). (**b**) ^13^C NMR spectrum from 180 to 120 ppm of **(NH_3_^+^)_8_[GMPA]-[MPA](C17)_4_** in CD_3_OD recorded at 100 MHz (full spectrum in Supplementary Materials, Figure S3). (**c**) FTIR spectra in transmission mode, (**d**) Mass spectrum of the *t*-Boc-protected dendrimer and (**e**) SEC chromatograms of the *t*-Boc-protected dendrimer and its two precursor dendrons.

The final Janus dendrimers were characterized by ^1^H NMR, ^13^C NMR and FTIR spectroscopy as well as Mass Spectrometry. The compound **(NH_3_^+^)_8_[GMPA]-[MPA](C17)_4_** is here presented as a representative example to discuss the characterization results (Figure 2). The correct click chemistry coupling was initially confirmed through ^1^H NMR (Figure 2a) by the presence of a peak at 8.07 ppm corresponding to proton *H-3* belonging to the triazole ring. Regarding the **(NH_3_^+^)_8_[GMPA]** block, the peaks corresponding to the protons *H-2* and *H-1* are shifted downfield in comparison with those in the starting dendron (from 3.27 to 4.45 ppm and from 1.61 to 1.95 ppm, respectively). With respect to the **[MPA](C17)_4_** block, the peak corresponding to the protons *H-5* is also shifted downfield (from 4.72 to 5.26 ppm) in comparison with the uncoupled dendron. The other signals remain essentially unaltered and a perfect correlation is observed for the signal integrations corresponding to the two blocks. In the ^13^C NMR spectrum, two peaks corresponding to the carbon atoms of the triazole *C-3* and *C-4*, at 126 and 144 ppm, respectively, are observed (Figure 2b). FTIR spectroscopy showed that bands corresponding to the stretching vibrations of the azide (–N=N^+^=N^–^, 2106 cm^–1^) and alkyne (≡C–H, 3292 cm^–1^ and the –C≡C–, 2141 cm^–1^) functional groups observed in the corresponding dendrons, before coupling, were not observed in the final Janus dendrimer, thus indicating the formation of the triazole ring (Figure 2c). The molecular peak of the dendrimer plus sodium was observed in the MS spectra (Figure 2d). Additionally, the monodispersity of the Janus dendrimers, the terminal amino groups of which were protected with *t*-Boc moieties, was confirmed by Size Exclusion Chromatography (SEC) showing a single and symmetrical monomodal peak (purple graph in Figure 2e). This technique allowed us to confirm the absence of unreacted dendrons **≡-[MPA](C17)_4_** (red graph in Figure 2e) and **N_3_-[GMPA](NHBoc)_8_** (blue graph in Figure 2e).

### 3.2. Preparation and Characterization of the Dendrimer Aggregates

#### 3.2.1. Formation of the Dendrimer Aggregates and Critical Aggregation Concentration (CAC) Determination

The formation of the dendrimer aggregates was achieved by the oil-in-water method as described in Section 2.2.2 Initially, we assessed the required concentration to ensure the presence of aggregates in water by determining the critical aggregation concentration (CAC) of all the dendrimers using nile red as a solvatochromic fluorophore [30]. The CAC for the studied Janus dendrimers is in the 10^−5^ M order of magnitude (Table 1 and Figure S4 in Supplementary Materials), which is consistent with related dendritic structures previously published [25].

#### 3.2.2. Morphological Studies of the Dendrimer Aggregates

**Figure 3:**
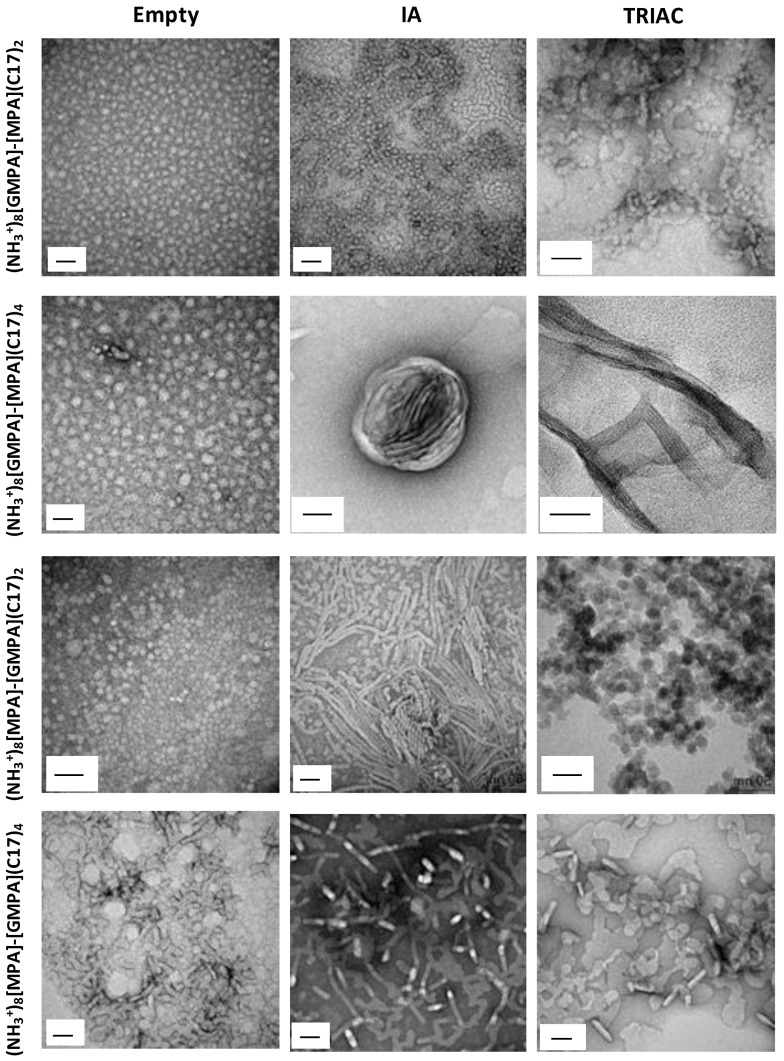
TEM images of the empty Janus dendrimer aggregates (first column) and loaded with IA (second column) or TRIAC (third column). Scale size: 50 nm.

The morphology of the aggregates formed by the amphiphilic Janus dendrimers in water was studied by TEM (Figure 3, left column) and their size (hydrodynamic diameter, D_H_) was determined by DLS. Dendrimers **(NH_3_^+^)_8_[GMPA]-[MPA](C17)_2_**, **(NH_3_^+^)_8_[GMPA]-[MPA](C17)_4_**, and **(NH_3_^+^)_8_[MPA]-[GMPA](C17)_2_** appeared as rounded micelles in TEM images, with a homogenous size distribution, and DLS measurements (Figure S5) gave number average D_H_ values of 9 ± 1, 24 ± 1 and 17 ± 3 nm, respectively. Longer aggregates resembling cylindrical micelles were observed in TEM for **(NH_3_^+^)_8_[MPA]-[GMPA](C17)_4_**, which gave number average D_H_ values of 34 ± 5 nm in DLS.

In order to relate the lipophilicity of the amphiphilic dendrimers to their assembly behavior in water, we calculated the lipophilic content Lc of these molecules as in our previous studies [25,38]. Here, we define Lc as the percentage in weight of the n-alkylic content in the entire dendrimer (Table 1). The two dendrimers with two stearate chains, **(NH_3_^+^)_8_[GMPA]-[MPA](C17)_2_** and **(NH_3_^+^)_8_[MPA]-[GMPA](C17)_2_**, show spherical morphology corresponding to similar and low Lc values of 9.7 and 10.7, respectively. On the other hand, both **(NH_3_^+^)_8_[GMPA]-[MPA](C17)_4_** and **(NH_3_^+^)_8_[MPA]-[GMPA](C17)_4_** form bigger aggregates and, interestingly, these two dendrimers assemble in a different morphology despite they display the same Lc (Lc = 14.8). In this case, the different position of the poly(esteramide) (GMPA) and polyester (MPA) dendrons with respect to the hydrophilic amino groups and lipophilic stearic acid chains seems to be responsible for the different micellar morphologies: spherical for (**NH_3_^+^)_8_[GMPA]-[MPA](C17)_4_** and cylindrical for (**NH_3_^+^)_8_[MPA]-[GMPA](C17)_4_**. This led us to suggest that the presence of amide groups in the GMPA dendron makes it more hydrophilic than MPA and hence their integration next to the hydrophilic outer ammonium groups enhances the hydrophilicity of this dendritic face of the Janus dendrimer. Accordingly, whereas **(NH_3_^+^)_8_[GMPA]-[MPA](C17)_4_** presents a well-defined Janus structure (with two demarcated hydrophilic and hydrophobic faces), **(NH_3_^+^)_8_[MPA]-[GMPA](C17)_4_** shows an alternated sequence hydrophilic ammonium-MPA-GMPA-hydrophobic C_17_H_35_ which results in a less defined Janus structure. This can disrupt the hydrophobic core of rounded micelles towards wormlike micelles with bigger space occupied by the inner part of the Janus molecules. This disrupting effect in the core of the spherical micelles is not as significant for **(NH_3_^+^)_8_[MPA]-[GMPA](C17)_2_,** but it could also explain why this dendrimer gives micelles almost twice in size than **(NH_3_^+^)_8_[GMPA]-[MPA](C17)_2_** in spite of their similar Lc and Mw.

#### 3.2.3. Cell Viability Studies in Huh 5-2 Cell Line

**Figure 4:**
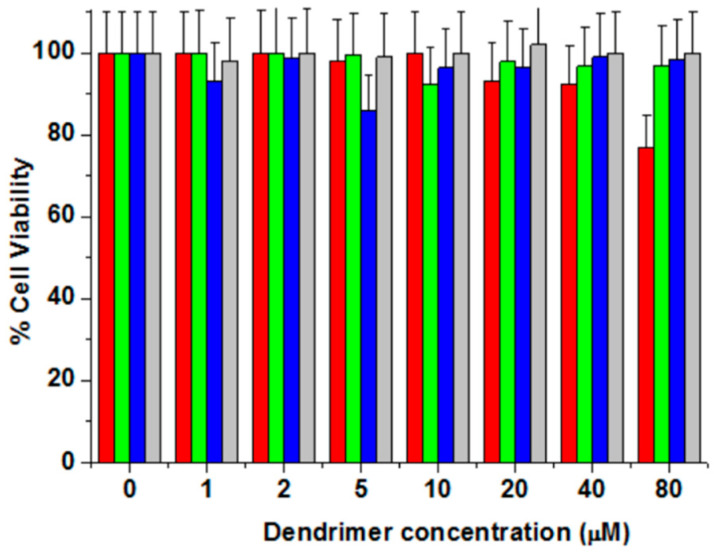
Cell Viability of empty Janus dendrimers in Huh 5-2 cell line. All the data are presented as the average ± standard deviation. **(NH_3_^+^)_8_[GMPA]-[MPA](C17)_2_** (Red Bars), **(NH_3_^+^)_8_[GMPA]-[MPA](C17)_4_** (Green Bars), **(NH_3_^+^)_8_[MPA]-[GMPA](C17)_2_** (Blue Bars), **(NH_3_^+^)_8_[MPA]-[GMPA](C17)_4_** (Gray Bars).

In order to confirm the suitability of the dendrimer aggregates to work as carriers for anti-HCV drugs, their cytotoxicity on the Huh5-2 cell line was studied (Figure 4). The four empty dendrimer aggregates showed cell viabilities larger than 85% up to a concentration 40 µM. Only the dendrimer **(NH_3_^+^)_8_[GMPA]-[MPA](C17)_2_** showed increased toxicity at 80 µM. These results indicate that all these dendrimers could be used as antiviral-drug carriers without compromising the cell viability.

### 3.3. Drug-Loaded Dendrimer Aggregates

#### 3.3.1. Isothermal Titration Calorimetry (ITC) Studies

**Figure 5:**
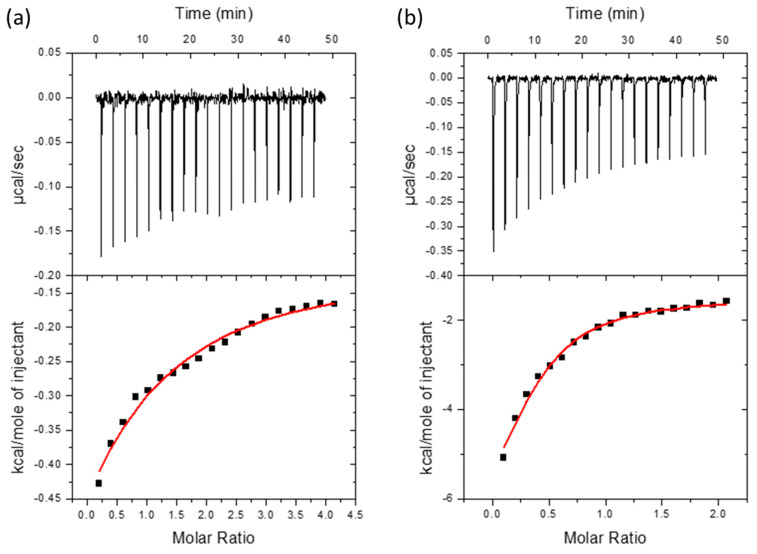
Interaction of compounds IA (**a**) and TRIAC (**b**) with **(NH_3_^+^)_8_[GMPA]-[MPA](C17)_2_** dendrimer aggregates assessed by ITC. Upper plots show the thermogram (raw thermal power as a function of time) and the lower plots show the binding isotherm (ligand-normalized heat effects as a function of the molar ratio). A single ligand binding site model has been used to fit the curve (in red).

In order to investigate the affinity of the dendrimer aggregates and IA and TRIAC molecules ITC experiments were performed. Thermograms and binding isotherms obtained for the (**(NH_3_^+^)_8_[GMPA]-[MPA](C17)_2_** dendrimer with IA and TRIAC are shown in Figure 5 as a representative example (data for the complete series can be found in Figure S6 of the Supplementary Materials). As shown in Table 2, we could confirm the interaction between each dendrimer and each of the drugs (ΔG from −5.2 to −8.2). All dendrimers tested bind TRIAC with similar affinity, with association constants (K_a_) in the range of 10^4^–10^5^ M^−1^, while affinity towards IA differs more with K_a_ ranging from 10^3^ to 10^6^ M^−1^. Furthermore, most of the systems studied exhibited a dendrimer-drug interaction mainly driven by entropic contributions (−T·ΔS), except for the **(NH_3_^+^)_8_[GMPA]-[MPA](C17)_2_** and **(NH_3_^+^)_8_[MPA]-[GMPA](C17)_4_** with TRIAC where enthalpic contributions have more importance in the resulting interaction (see also Figure S7 of the Supplementary Materials).

#### 3.3.2. Drug Loading

The lipophilic IA and TRIAC drugs were loaded within the dendrimer aggregates using the solvent diffusion technique, where DMSO was used as a water miscible co-solvent of the dendrimer aggregates and the drug. Minimal amounts of DMSO, namely 2.5% (*v*/*v*), were added in order not to interfere with the stability of the aggregates. An excess of drug was used to maximize its loading within the dendrimer aggregates. Namely, the drug and dendrimer were mixed in a ratio of 5/1 (mol_drug_/mol_dend_) for 16 h. A white precipitate appeared during the dialysis that corresponded to non-trapped drug, which was removed by filtration with 0.22 μm syringe filters. The amount of encapsulated drug was directly measured by UV-Visible absorbance by disrupting the loaded nanocarriers with a high amount of DMSO (75% in volume) and the molar ratio between the loaded drug and dendrimer (drug loading content, DLC) for each nanocarrier was calculated (Table 1).

DLC values between 1.4 and 1.9 were obtained for both drugs within **(NH_3_^+^)_8_[GMPA]-[MPA](C17)_2_**, **(NH_3_^+^)_8_[GMPA]-[MPA](C17)_4_**, and **(NH_3_^+^)_8_[MPA]-[GMPA](C17)_2_**, while **(NH_3_^+^)_8_[MPA]-[GMPA](C17)_4_** exhibited poor loading skills. Non-significant differences were observed between IA and TRIAC DLC values. Considering that the encapsulation takes place by diffusion once the dendrimer aggregates are already formed, their initial morphology seems to be relevant during the process. In this regard, while the empty aggregates of **(NH_3_^+^)_8_[GMPA]-[MPA](C17)_2_**, **(NH_3_^+^)_8_[GMPA]-[MPA](C17)_4_**, and **(NH_3_^+^)_8_[MPA]-[GMPA](C17)_2_** exhibited rounded morphology, **(NH_3_^+^)_8_[MPA]-[GMPA](C17)_4_** presented a cylindrical morphology. We initially considered that the amide groups present in the GMPA dendron could facilitate the encapsulation by the establishment of hydrogen bonds with the drugs. Indeed, the inclusion of this dendritic scaffold permits suitable DLC values for antiviral activity studies, regardless its position in the Janus structure for the three dendrimers providing spherical micelles. However, this is not the case for the **(NH_3_^+^)_8_[MPA]-[GMPA](C17)_4_** dendrimer that shows poor encapsulation properties. As a tentative explanation, we propose that the cylindrical shape observed for its aggregates could hamper the accessibility of the drugs to the GMPA dendron, which is located closer to the core of the nanostructure. Besides, the size of the hydrophilic face has been described in other studies to stabilize the dendrimer/drug aggregates once a lipophilic drug is included [25]. Then, as mentioned above, the lower hydrophilicity and less-defined Janus structure displayed by **(NH_3_^+^)_8_[MPA]-[GMPA](C17)_4_**, may also increase the instability in water of the dendrimer/drug aggregates, which can also contribute to explain the poor DLC values observed. Although the decrease is not as dramatic, **(NH_3_^+^)_8_[GMPA]-[MPA](C17)_4_** shows also lower DLC than the Janus with the smallest lipophilic dendrons. It seems that the biggest lipophilic dendrons do not benefit the loading of the highest amount of any of these drugs. Similar observations were obtained for CPT, an anti-HCV lipophilic drug previously assayed by encapsulation on bis-MPA-based amphiphilic Janus dendrimers [25]. The inclusion of lipophilic molecules alters the hydrophilic/lipophilic balance of the dendrimer/drug aggregates determining the amount of drug loaded, and this effect is more significant for lower hydrophilic/lipophilic balances, i.e., higher Lc.

The morphology of the aggregates after drug loading was studied by TEM as shown in Figure 3, middle and right columns for IA and TRIAC derivatives, respectively. The loading of the compounds led to significant morphological changes in **(NH_3_^+^)_8_[GMPA]-[MPA](C17)_2_**, **(NH_3_^+^)_8_[GMPA]-[MPA](C17)_4_**, and **(NH_3_^+^)_8_[MPA]-[GMPA](C17)_2_**, whereas the morphology of **(NH_3_^+^)_8_[MPA]-[GMPA](C17)_4_** remained almost unchanged after the diffusion process. The changes observed for **(NH_3_^+^)_8_[GMPA]-[MPA](C17)_2_/**drug (IA and TRIAC) aggregates seem related with aggregation effects of spherical micelles. No significant shape disruptions were detected, and this is in agreement with the stabilizing role of its bigger hydrophilic dendron, **(NH_3_^+^)_8_[GMPA],** even after the increase of lipophilicity provoked by the addition of the drug. In contrast, the inclusion of each drug within the **(NH_3_^+^)_8_[GMPA]-[MPA](C17)_4_** aggregates turned into the most drastic morphological changes and elongated aggregates were observed. The formation of wormlike micelles for **(NH_3_^+^)_8_[GMPA]-[MPA](C17)_4_**/IA and long cylindrical micelles for **(NH_3_^+^)_8_[GMPA]-[MPA](C17)_4_**/TRIAC can indeed be associated with a significant increase of the lipophilic contents, which is not compensated by the size of the hydrophilic dendron [38,39,40]. Moreover, such elongated aggregates self-arrange into large assemblies. Upon IA loading, the wormlike micelles rolled up into large spherical assemblies (see also Figure S9), and the TRIAC-loaded long cylindrical micelles longitudinally arrange in lamellar structures. The formation of wormlike micelles was also detected for **(NH_3_^+^)_8_[MPA]-[GMPA](C17)_2_** loaded with IA, although in this case the micelles appeared more dispersed. Upon TRIAC loading, **(NH_3_^+^)_8_[MPA]-[GMPA](C17)_2_** aggregates maintained the spherical morphology. In contrast, the drug loading process carried out for **(NH_3_^+^)_8_[MPA]-[GMPA](C17)_4_** only slightly affected the morphological characteristics of the aggregates, and this is consistent with the low DLC values measured (Table 1).

### 3.4. In Vitro Antiviral Activity and Cell Viability of the Dendrimer/Drug Aggregates

The efficacy of the **(NH_3_^+^)_8_[GMPA]-[MPA](C17)_2_**, **(NH_3_^+^)_8_[GMPA]-[MPA](C17)_4_**, and **(NH_3_^+^)_8_[MPA]-[GMPA](C17)_2_** as nanocarriers for IA or TRIAC to inhibit the replication rate of HCV replicon was measured together with their cell viability in the optimized Huh 5-2 cells containing subgenomic HCV replicons. Considering the low loading ability of **(NH_3_^+^)_8_[MPA]-[GMPA](C17)_4_**, this dendrimer was not included in these experiments. For each drug, the cell viability and viral replication inhibition of the three dendrimer/drug aggregates were measured at increasing drug concentrations and compared with the corresponding free drug (Table 3).

**Figure 6:**
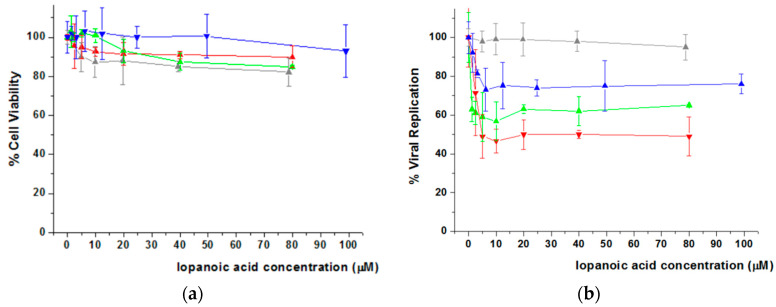
Cell Viability (**a**) and HCV viral replication (**b**) of free and IA-loaded Janus dendrimers in Huh 5-2 cell line. All the data are presented as the average ± standard deviation. Free IA (Grey Line), **(NH_3_^+^)_8_[GMPA]-[MPA](C17)_2_/IA** (Red Line) **(NH_3_^+^)_8_[GMPA]-[MPA](C17)_4_/IA** (Green Line) **(NH_3_^+^)_8_[MPA]-[GMPA](C17)_2_/IA** (Blue Line). For the sake of the clarity, the highest concentration of IA within **(NH_3_^+^)_8_[GMPA]-[MPA](C17)_2_** (i.e., 160 μM) is not represented neither for cell viability nor for viral replication inhibition. The full plots are represented in Figure S11 of the Supplementary Materials.

Figure 6 shows the results obtained for IA. The percentage of cell viability measured for each dendrimer/IA aggregate and free IA is represented in Figure 6a. It is observed that the three systems seem to be biocompatible as cell viability remained above 80% at the highest drug concentrations tested (Figure S11). Regarding antiviral activity (Figure 6b), all the IA-loaded derivatives enhanced the inhibition of viral replication in comparison with the free drug. In particular, those containing the *bis*-GMPA hydrophilic dendron in their structure, i.e., **(NH_3_^+^)_8_[GMPA]-[MPA](C17)_2_** and **(NH_3_^+^)_8_[GMPA]-[MPA](C17)_4_**, showed the highest inhibition values (lower IA concentration was needed to inhibit 40% of viral replication).

**Figure 7:**
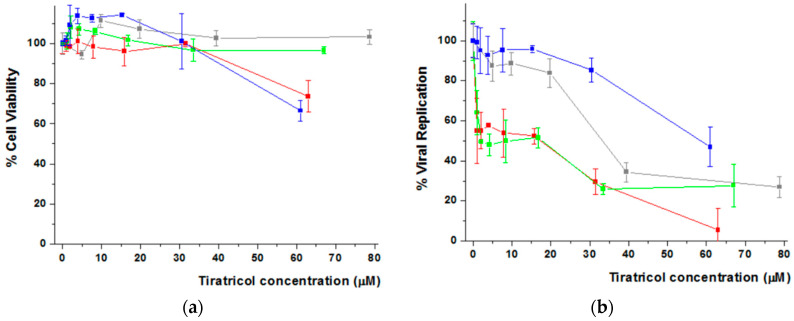
Cell Viability (**a**) and HCV viral replication (**b**) of Free and loaded TRIAC in Janus dendrimers in Huh 5-2 cell line. All the data are presented as the average ± standard deviation. Free TRIAC (Grey Line) **(NH_3_^+^)_8_[GMPA]-[MPA](C17)_2_/TRIAC** (Red Line) **(NH_3_^+^)_8_[GMPA]-[MPA](C17)_4_/TRIAC** (Green Line) **(NH_3_^+^)_8_[MPA]-[GMPA](C17)_2_/TRIAC** (Blue Line).

Cell treatment with TRIAC loaded dendrimer aggregates did not show a decrease of initial cell viability (Figure 7a), and viral replication studies (Figure 7b) revealed that **(NH_3_^+^)_8_[GMPA]-[MPA](C17)_2_** and **(NH_3_^+^)_8_[GMPA]-[MPA](C17)_4_** significantly improved the activity of the free drug, reaching EC40 at concentrations of TRIAC lower than 5 and 2.5 μM respectively, which means 6 or 12 times lower, respectively, compared with EC40 of free TRIAC (20–40 μM). With respect to viability, none of both systems showed cytotoxic effect up to drug concentrations of 30 μM. Surprisingly, whereas **(NH_3_^+^)_8_[GMPA]-[MPA](C17)_4_/TRIAC** did not show toxicity within the full drug-concentration range studied, **(NH_3_^+^)_8_[GMPA]-[MPA](C17)_2_/TRIAC** displayed decreased cell viability at the highest TRIAC concentration tested, i.e., 60 μM, which must be related with the increase of toxicity observed for the dendrimer itself at the highest concentration (Figure 4). In any case, this does not detract its potential as effective nanocarrier given its capacity to decrease by a factor of 6 the EC40 of the free drug. In contrast to dendrimers with *bis*-MPA at the lipophilic dendron, **(NH_3_^+^)_8_[MPA]-[GMPA](C17)_2_** did not provide a valid carrier system with an EC40 value, i.e., 60 μM, even higher than free TRIAC. Such an inhibition effect is likely due to a significant cytotoxic effect, which appears for **(NH_3_^+^)_8_[MPA]-[GMPA](C17)_2_/TRIAC** at a TRIAC concentration at which the free drug is fully biocompatible.

### 3.5. Cellular Internalization Studies by Flow Cytometry Imaging

**Figure 8:**
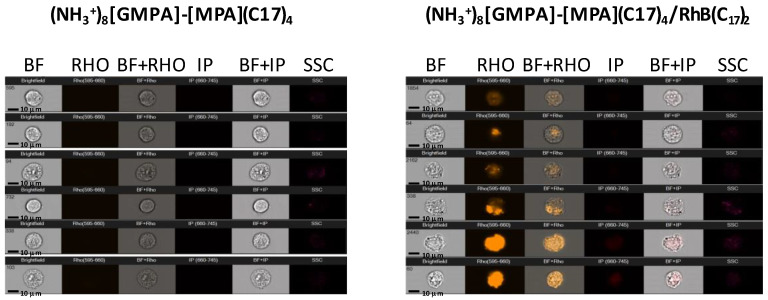
Examples of AMNIS cytometer cell images. Huh 5-2 cells treated with **(NH_3_^+^)_8_[GMPA]-[MPA](C17)_4_** (**left**) **(NH_3_^+^)_8_[GMPA]-[MPA](C17)_4_/RhB(C17)_2_** (**right**). Each column collects images taken under different light sources. BF (brightfield), Rho (Rhodamine fluorescence was measured by exciting at 488 nm and detecting emission at 529 nm using AMNIS cytometer); IP (Propidium iodide was excited using a laser that emits at 488 nm and emitted fluorescence was detected at 660 nm in another channel to avoid emission fluorescence overlap rhodamine); SSC (side scattered channel).

In order to explore the ability of the dendrimer/drug aggregates to carry its cargo into the cell, internalization experiments were performed using an ImageStream X AMNIS Morphocytometric System, which combines high-speed microscopic image capture with flow cytometry. For this study, **(NH_3_^+^)_8_[GMPA]-[MPA](C17)_4_** was selected and an amphiphilic rhodamine derivative, *RhB(C17)_2_* (Figure S12), was used as a fluorescent probe [41]. Figure 8 displays the selected microscope images that allowed us to detect the internalization of the fluorescent cargo in single cells. Using the AMNIS System, not only visualization but also quantification is possible through flow cytometry analysis. Rhodamine fluorescence was detected within the cells and the percentage of counted cells was over 67.3% in the case of **(NH_3_^+^)_8_[GMPA]-[MPA](C17)_4_/RhB(C17)_2_**, in contrast to negative controls and bare dendrimer aggregates that did not show fluorescence. Furthermore, the cellular cycle was not altered upon treatment neither with **(NH_3_^+^)_8_[GMPA]-[MPA](C17)_4_** nor with **(NH_3_^+^)_8_[GMPA]-[MPA](C17)_4_/RhB(C17)_2_** assessed at 3.7 μM (concentration above the one required to reach the EC40 of both drugs in the drug-loaded dendrimer aggregates), thus confirming that this dendrimer was not toxic by itself.

## 4. Conclusions

The present study shows the ability of amphiphilic Janus dendrimers to provide effective nanocarriers for IA and TRIAC, two anti-HCV drugs that were identified as HCV NS3 protease allosteric inhibitors in primary screenings. The combination through click chemistry of ammonium-terminated with stearic acid-terminated dendrons, derived from *bis*-GMPA and *bis*-MPA moieties, afforded a series of four novel amphiphilic Janus with different lipophilic content and alternation of polyester (MPA) and poly(esteramide) (GMPA) dendritic blocks. TEM images showed a distortion of rounded morphologies towards cylindrical micelles and this seems to be related with the different hydrophilic nature of the GMPA dendrons versus MPA dendrons. Thus, the generation and the number of the lipophilic blocks, together with the different alternated positions of MPA and GMPA dendrons in the architecture of the Janus dendrimers, influence not only the morphology but also the drug-loading ability of the dendrimer aggregates. Whereas **(NH_3_^+^)_8_[GMPA]-[MPA](C17)_2_, (NH_3_^+^)_8_[GMPA]-[MPA](C17)_4_**, and **(NH_3_^+^)_8_[MPA]-[GMPA](C17)_2_** show high drug loading contents for both IA and TRIAC drugs, **(NH_3_^+^)_8_[MPA]-[GMPA](C17)_4_** did not display good loading capacity, and this ruled out its interest as an effective nanocarrier. Attending antiviral activity assays into Huh 5-2 cells, EC40 values of the studied drugs were significantly reduced when encapsulated within the **(NH_3_^+^)_8_[GMPA]-[MPA](C17)_2_** and **(NH_3_^+^)_8_[GMPA]-[MPA](C17)_4_** aggregates, whereas **(NH_3_^+^)_8_[MPA]-[GMPA](C17)_2_** was discarded as a good nanocarrier against hepatitis C.

Overall, it can be concluded that dendrimers containing the poly(esteramide), *bis*-GMPA, architecture in the hydrophilic side yield the nanocarriers with better performances for the studied drugs, being **(NH_3_^+^)_8_[GMPA]-[MPA](C17)_2_/IA** and **(NH_3_^+^)_8_[GMPA]-[MPA](C17)_4_/TRIAC** the best antiviral combinations as far as their lack of cytotoxicity and inhibitory activity of HCV replication are concerned.

## Figures and Tables

**Table 1:** Lc and critical aggregation concentration (CAC) values for the amphiphilic Janus dendrimers, number average hydrodynamic diameters, D_H_, of the dendritic aggregates measured by DLS and amount of IA and TRIAC loaded within the Janus dendrimers.

Dendrimer				IA		TRIAC	
	Lc ^a^ (%)	CAC (M)	D_H_ (nm)	[IA] (μM)	*DLC*^c^ (mol/mol)	[TRIAC] (μM)	*DLC*^c^ (mol/mol)
**(NH_3_^+^)_8_[GMPA]-[MPA](C17)_2_**	9.7	1.1·10^−5^	9 ± 1	680 ± 172	1.86	563 ± 257	1.65
**(NH_3_^+^)_8_[GMPA]-[MPA](C17)_4_**	14.8	6.9·10^−6^	24 ± 1	321 ± 119	1.44	505 ± 238	1.59
**(NH_3_^+^)_8_[MPA]-[GMPA](C17)_2_**	10.7	8.1·10^−6^	17 ± 3	641 ± 207	1.78	583 ± 167	1.81
**(NH_3_^+^)_8_[MPA]-[GMPA](C17)_4_**	14.8	1.2·10^−5^	34 ± 5 ^b^	35 ± 16	0.19	70 ± 49	0.29

^a^ Lc was calculated by dividing the MW of the lipophilic dendron by the MW of the Janus amphiphilic dendrimer and multiplied by 100. ^b^ **(NH_3_^+^)_8_[MPA]-[GMPA](C17)_4_** appears as cylindrical micelles in TEM images. ^c^ DLC was calculated as the molar ratio between the amount of drug entrapped as determined by UV/Vis spectroscopy and the amount of Janus dendrimer.

**Table 2:** Thermodynamic parameters calculated from ITC assays for the interaction between the Janus dendrimers and the compounds IA and TRIAC. K_a_ values are expressed in M^−1^ and ΔG, ΔH and (−T·ΔS) are expressed in kcal/mol.

		K_a_	ΔG	ΔH	−T·ΔS
**(NH_3_^+^)_8_[GMPA]-[MPA](C17)_2_**	**IA**	6.3 × 10^3^	−5.2	−1.1	−4.1
	**TRIAC**	1.0 × 10^5^	−6.8	−6.0	−0.8
**(NH_3_^+^)_8_[GMPA]-[MPA](C17)_4_**	**IA**	9.2 × 10^4^	−6.8	−0.4	−6.4
	**TRIAC**	6.1 × 10^5^	−7.9	−0.4	−7.5
**(NH_3_^+^)_8_[MPA]-[GMPA](C17)_2_**	**IA**	4.8 × 10^4^	−6.4	−0.1	−6.3
	**TRIAC**	1.5 × 10^5^	−7.1	−1.3	−5.8
**(NH_3_^+^)_8_[MPA]-[GMPA](C17)_4_**	**IA**	1.1 × 10^6^	−8.2	0.7	−8.9
	**TRIAC**	8.8 × 10^4^	−6.7	−15.8	9.1

Typical error in Ka is 30%, absolute error in ΔG is 0.2 kcal/mol, absolute errors in ΔH and −T·ΔS are 0.5 kcal/mol.

**Table 3:** CC50 and EC40 values for the dendrimer/drug aggregates compared to free drug values.

	[IA] (μM)		[TRIAC] (μM)	
	CC50	EC40	CC50	EC40
**(NH_3_^+^)_8_[GMPA]-[MPA](C17)_2_**	>160	2.5–5	>63	1
**(NH_3_^+^)_8_[GMPA]-[MPA](C17)_4_**	>80	2.5	>67	1
**(NH_3_^+^)_8_[MPA]-[GMPA](C17)_2_**	>99	>99	>61	30–60
**free drug**	300	175	188	20–40

## Supplementary Materials

The following are available online at https://www.mdpi.com/1999-4923/12/11/1062/s1, Scheme S1. Synthetic steps for the synthesis of the lipophilic dendrons; Figure S1 Chemical characterization of **≡-[GMPA](C17)_2_**: (a) 1H NMR, (b) ^13^C NMR, (c) FTIR spectrum in transmission mode and (d) MS spectrum; Figure S2. Chemical characterization of **≡-[GMPA](C17)_4_**: (a) 1H NMR, (b) ^13^C NMR, (c) FTIR spectrum in transmission mode and (d) MS spectrum. Figure S3. Full ^13^C NMR spectrum of dendrimer **(NH_3_^+^)_8_[GMPA]-[MPA](C17)_4_**. Figure S4. Determination of the CAC of all the Janus dendrimers. Figure S5. Size distribution measured by DLS. Figure S6. Interaction of drugs IA (a) and TRIAC (b) with **(NH_3_^+^)_8_[GMPA]-[MPA](C17)_4_**, **(NH_3_^+^)_8_[MPA]-[GMPA](C17)_2_** and **(NH_3_^+^)_8_[MPA]-[GMPA](C17)_4_** Janus dendrimer aggregates assessed by isothermal titration calorimetry (ITC). The upper plots in (a) and (b) show the thermogram (thermal power required to maintain a null temperature difference between sample and reference cells as a function of time) and the lower plots in A and B show the binding isotherm (ligand-normalized heat effect per injection as a function of the molar ratio, the quotient between the ligand and dendrimer concentrations in the cell). The fitting curve (in red) corresponds to the single ligand binding site model (continuous line); Figure S7. Graphical representation of thermodynamic parameters calculated from ITC assays for the interaction between Janus dendrimers and the compounds IA and TRIAC (extracted from Table 2). ΔG (blue bars), ΔH (red bars) and −T∙ΔS (green bars) are expressed in kcal/mol; Figure S8. Calibration curves used for the determination of IA and TRIAC concentrations; Figure S9. Additional TEM images of the **(NH_3_^+^)_8_[GMPA]-[MPA](C17)_4_** aggregates loaded with IA. Scale bar ranging from 200 to 50 nm; Figure S10. HCV viral replication of the Janus dendrimers in Huh 5-2 cell line. All the data are presented as the average ± standard deviation. **(NH_3_^+^)_8_ [GMPA]-[MPA](C17)_2_** (Red Line) **(NH_3_^+^)_8_ [GMPA]-[MPA](C17)_4_** (Green Line) and **(NH_3_^+^)_8_ [MPA]-[GMPA](C17)_2_** (Blue Line). Figure S11. Cell viability (line) and HCV viral replication (bars) of **(NH_3_^+^)_8_ [GMPA]-[MPA](C17)_2_/IA** in Huh 5-2 cell line including the highest concentration assayed, i.e., 160 μM. All the data are presented as the average ± standard deviation; Figure S12. Chemical structure of RhB(C17)2.
