# Supplementary material for: Janus Dendrimers to Assess the Anti-HCV Activity of Molecules in Cell-Assays
Source: Pharmaceutics. 2020 Nov 7;12(11):1062. doi: 10.3390/pharmaceutics12111062 (PMC7695217; doi:10.3390/pharmaceutics12111062)
Supplement: Supplementary file 1 [file pharmaceutics-12-01062-s001.pdf]

Supplementary Materials: Janus Dendrimers to Assess the Anti-HCV Activity of Molecules in Cell-Assays

María San Anselmo, Alexandre Lancelot, Julia Egido, Rafael Clavería-Gimeno, Álvaro Casanova, José Luis Serrano, Silvia Hernández-Ainsa, Olga Abian and Teresa Sierra

1. Synthesis of the dendrons ≡-[GMPA](C17)$_2$ and ≡-[GMPA](C17)$_4$:

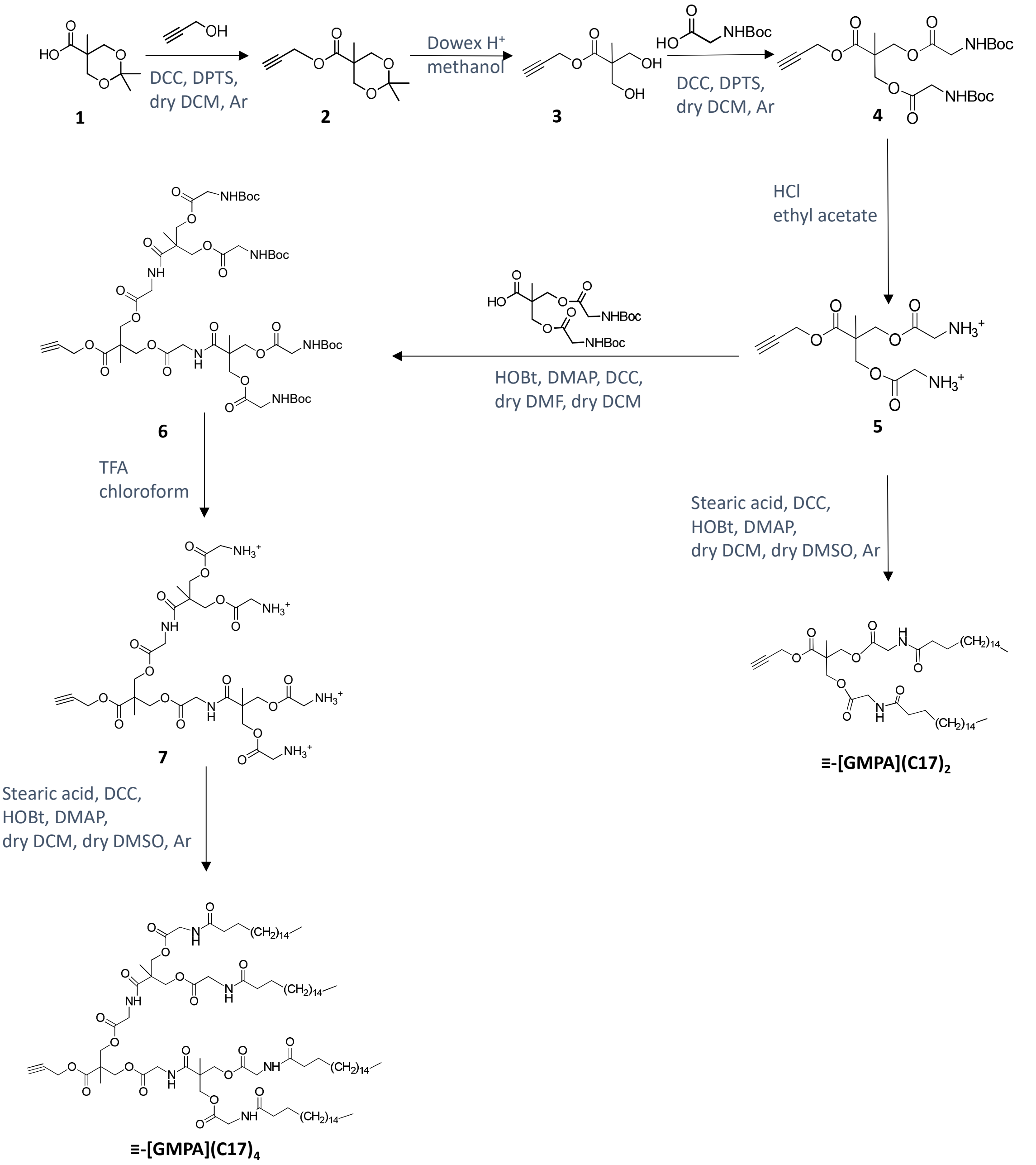

Scheme S1. Synthetic steps for the synthesis of the lipophilic dendrons ≡-[GMPA](C17)$_2$ and ≡-[GMPA](C17)$_4$.

pharmaceutics

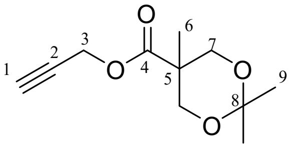

Compound 2. Propargyl alcohol (3.23 mL, 55.80 mmol, 1.05 eq.) was dissolved into dry DCM (80 mL). Acetonide protected *bis*-MPA (compound **1**) [1] (10.00 g, 53.10 mmol, 1.00 eq.) and DPTS (6.24 g, 21.20 mmol, 0.40 eq.) were added. The reaction mixture was stirred under argon atmosphere and was cooled down to 0 °C. A solution of DCC (10.96 g, 53.10 mmol, 1.05 eq.) in dry DCM (20 mL) was added dropwise. The reaction mixture was allowed to stir under argon atmosphere overnight at room temperature. The white precipitate, DCU, was filtered off and the solvent was evaporated under vacuum to get a mixture of oil and solid. DCU was newly precipitated into hexane and filtered off. The solvent was evaporated under reduced pressure. The crude product was purified on silica gel (hexane: ethyl acetate = 7:3) to give a light grey oil (10.77 g, 90 %). ^{1}H NMR (400 MHz, $CDCl_3$) δ (ppm): 4.71 (d, J = 2.4 Hz, 2H, H-3), 4.17 (d, J = 12 Hz, 2H, H-7), 3.63 (d, J = 12 Hz, 2H, H-7′), 2.46 (t, J = 2.4 Hz, 1H, H-1), 1.40 (d, 3H, H-9), 1.36 (s, 3H, H-9′), 1.18 (s, 3H, H-6). ^{13}C NMR (100 MHz, $CDCl_3$) δ (ppm): 173.3, 98.0, 77.4, 74.9, 65.8, 52.3, 41.8, 24.5, 22.5, 18.3. MS (ESI$^+$) *m*/*z* (%): found *235.0 (13),* calculated for $[C_{11}H_{16}O_4,Na]^+$ *235.1*. FTIR (ν_{max}/cm^{-1}, nujol): 3273 (≡C-H st), 2993 (C-H st), 2129 (C≡C st), 1739 (C=O st), 1456 (CH_2, CH_3 δ).

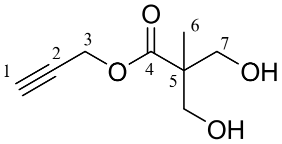

Compound 3. Dowex 50 WX2 hydrogen form (50-100 mesh) resin was washed by stirring in MeOH and was recovered by filtration. Compound **2** (10.55 g, 49.71 mmol, 1.00 eq.) was dissolved into MeOH (150 mL). Washed Dowex resin H^+ (5.22 g, 50% in weight) was added and the reaction was stirred for 5 hours. The resin was filtered off and the solvent was evaporated under vacuum to obtain a colorless oil (8.47 g, 99 %). ^{1}H NMR (400 MHz, $CDCl_3$) δ (ppm): 4.74 (d, J = 2.4 Hz, 2H, H-3), 3.90 (d, J = 10.6 Hz, 2H, H-7), 3.71 (d, J = 10.6 Hz, 2H, H-7′), 3.04 (bs, 2H, ~OH), 2.50 (t, J = 2.4 Hz, 1H, H-1), 1.09 (s, 3H, H-6). ^{13}C NMR (100 MHz, $CDCl_3$) δ (ppm): 175.0, 77.3, 75.2, 67.6, 52.4, 49.3, 17.0. MS (ESI$^+$) *m*/*z (%)*: found *194.9 (100)*, calculated for $[C_8H_{12}O_4,Na]^+$ *195.1*. FTIR (ν_{max}/cm^{-1}, nujol): 3293 (O-H st), 2945 (C-H st), 2128 (C≡C st), 1730 (C=O st), 1461 (CH_2, CH_3 δ).

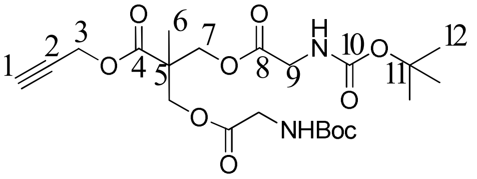

Compound 4. Compound **3** (8.00 g, 46.46 mmol, 1.00 eq.) was dissolved into dry DCM (220 mL), together with *N*-(*tert*-butoxycarbonyl)glycine (20.35 g, 116.15 mmol, 2.50 eq.), DPTS (6.70 g, 22.76 mmol, 0.50 eq.) and DMAP (3.70 g, 30.29 mmol, 0.70 eq.). This mixture was stirred under argon atmosphere and cooled down to 0 °C. A solution of DCC (23.97 g, 116.15 mmol, 2.50 eq.) in dry DCM (30 mL) was added dropwise. The reaction mixture was then allowed to stir under argon atmosphere overnight at room temperature. A white precipitate, DCU, appeared and was removed by filtration. The solvent of the resulting mixture was evaporated under vacuum. A mixture of hexane and ethyl acetate (4:1) was added to provoke further precipitation of the DCU, which was filtered off. The solvent was evaporated under reduced pressure. The crude product was purified through silica gel column chromatography (hexane: ethyl acetate ramp from 9:1 to 7:3). A yellow sticky solid was obtained (19.10 g, 85 %). ^{1}H NMR (400 MHz, $CDCl_3$) δ (ppm): 5.19 – 5.11 (bs, 2H, -NH), 4.69 (d, *J* = 2.5 Hz, 2H, H-3), 4.28 (dd, J_1 = 26.4, J_2 = 11.1 Hz, 4H, H-7), 3.86 (d, *J* = 5.9 Hz, 4H, H-9), 2.50 (t, *J* = 2.4 Hz, 1H, H-1), 1.40 (s, 18H, H-12), 1.25 (s, 3H, H-6). ^{13}C NMR (100 MHz, $CDCl_3$) δ (ppm): 171.7, 170.1, 155.8, 80.1, 77.5, 75.5, 65.7, 52.8, 46.4, 42.3, 28.4, 17.9. MS (ESI$^+$) *m/z (%)*: found *509.0 (100)*, calculated for $[C_{22}H_{34}N_2O_{10},Na]^+$ *509.5*. FTIR (ν_{max}/cm^{-1}, ATR): 3383 (N-H st), 3283 (≡C-H st), 2980 and 2939 (C-H st), 2127 (C≡C st), 1744 (C=O st ester), 1699 (C=O st carbamate), 1514 (N-H δ), 1367 (C-N st), 1248 (COO st), 1155 (O-C-C st).

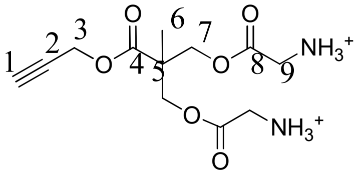

Compound 5. *Deprotection method A described in section 2.1.3 in manuscript.* Compound **4** (9.98 g, 20.51 mmol, 1.00 eq.). The final product was obtained as a white powder (5.74 g, 78 %). ^{1}H NMR (400 MHz, $(CD_3)_2SO$) δ (ppm): 8.56 (bs, -NH), 4.73 (s, 2H, H-3), 4.32 (s, 4H, H-7), 3.77 (s, 4H, H-9), 3.43 (s, 1H, H-1), 1.22 (s, 3H, H-6). ^{13}C NMR (100 MHz, $(CD_3)_2SO$) δ (ppm): 171.2, 167.1, 78.2, 78.0, 65.8, 52.8, 45.9, 39.5, 17.2. MS (ESI$^+$) *m/z (%)*: found *286.9 (100)*, calculated for $[C_{12}H_{18}N_2O_6,H]^+$ *287.1*. FTIR (ν_{max}/cm^{-1}, ATR): 3300 - 2560 (bs N-H$^+$ st), 1761 and 1726 (C=O st ester), 1555 (N-H$^+$ δ), 1502 (CH_2, CH_3 δ), 1406 (C-N st), 1170 (O-C-C st).

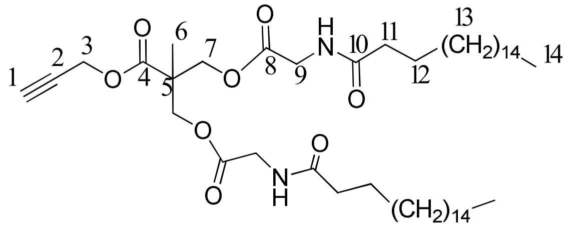

≡-[GMPA](C17)$_2$. Stearic acid (2.37 g, 8.34 mmol, 3.00 eq.), DMAP (0.27 g, 2.22 mmol, 0.80 eq.), HOBt (1.28 g, 8.34 mmol, 3.00 eq.) and DCC (1.72 g, 8.34 mmol, 3.00 eq.) were dissolved in dry DCM (90 mL). Compound **5** (1.00 g, 2.78 mmol, 1.00 eq.) and DMAP (0.68 g, 5.56 mmol, 2.00 eq.) were dissolved in anhydrous DMSO (10 mL) and was added dropwise to the afore-mentioned solution using a dropping funnel. The final reaction mixture was stirred at room temperature under argon atmosphere for 48 hours. A white precipitate appeared, DCU, which was filtered off. The solvent was then evaporated under vacuum. The crude product was dissolved into the minimal volume of DCM and purified by precipitation into cold acetone overnight. The precipitate was further purified through silica gel column chromatography (DCM: MeOH = 98: 2). Finally, a brown solid was obtained (1.86 g, 82 %). ^{1}H NMR (400 MHz, $CDCl_3$) δ (ppm): 6.16 (bs, -NH), 4.72 (d, J = 2.4 Hz, 2H, H-3), 4.32 (m, 4H, H-7), 4.01 (d, J = 5.4 Hz, 4H, H-9), 2.50 (t, J = 2.4 Hz, 1H, H-1), 2.24 (t, 4H, H-11), 1.68 – 1.57 (m, 4H, H-12), 1.28 (s, 3H, H-6), 1.24 (m, 56H, H-13), 0.87 (t, *J* = 6.8 Hz, 6H, H-14). ^{13}C NMR (100 MHz, $CDCl_3$) δ (ppm): 173.7, 171.8, 169.8, 75.6, 65.8, 52.9, 46.3, 41.2, 36.5, 32.1, [29.9-29.3], 25.7, 22.8, 18.2, 14.3. MS (ESI$^+$) *m/z (%)*: found *841.5 (100)*, calculated for $[C_{48}H_{86}N_2O_8,Na]^+$ *841.6*. FTIR (ν_{max}/cm^{-1}, ATR): 3294 (N-H st), 2955 and 2916 (C-H st), 1744 (C=O ester st), 1645 (C=O amide st), 1555 (N-H δ), 1470 (CH_2, CH_3 δ), 1203 (C-O st). Spectra are shown in figure S1.

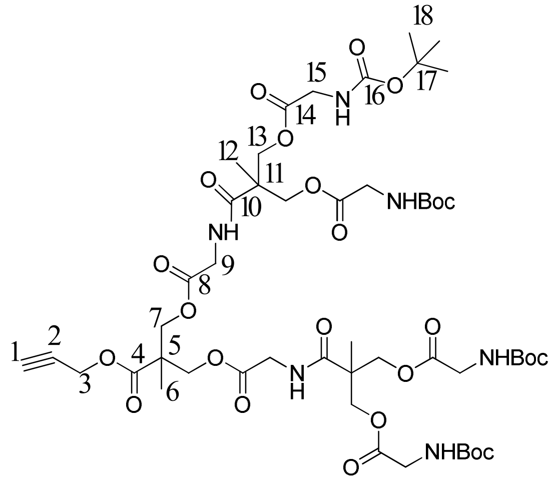

Compound 6. Compound **5** (2.00 g, 5.57 mmol, 1.00 eq.) was dissolved into dry DMF. 2,2'-*bis*(glyciloxy)propionic acid [1] (5.49 g, 12.25 mmol, 2.20 eq.) was dissolved into dry DCM. Both were mixed and then HOBt (1.66 g, 12.25 mmol, 2.20 eq.) and DMAP (1.91 g, 15.59 mmol, 2.80 eq.) were added and stirred under argon atmosphere, cooling the mixture down to 0 °C. DCC (2.53 g, 12.25 mmol, 2.20 eq.) was dissolved into dry DCM and was added dropwise to the reaction mixture. It was allowed to stir at room temperature under argon atmosphere for 5 days. The white precipitate which appeared, DCU, was filtered off and the solvent was evaporated under vacuum. After dissolving in ethyl acetate, the organic phase was washed three times with brine (in order to eliminate the remaining DMF), dried over anhydrous $MgSO_4$ and the solvent was evaporated under vacuum. The orange oil obtained was purified by silica gel chromatography (hexane: ethyl acetate, 3: 7) to get a white powder (3.44 g, 54 %). ^{1}H NMR (400 MHz, $CDCl_3$) δ (ppm): 7.09 (bs, -NHCO), 5.29 (bs, -NHBoc), 4.73 (d, J = 2.4 Hz, 2H, H-3), [4.45-4.19] (m, 12H, H-7, H-13), 3.98 (d, J = 5.4 Hz, 4H, H-9), 3.91 (d, J = 5.7 Hz, 8H, H-15), 2.52 (t, J = 2.4 Hz, 1H, H-1), 1.43 (s, 36H, H-18), 1.28 (s, 9H, H-6, H-12). ^{13}C NMR (100 MHz, CD_3OD) δ (ppm): 175.3, 173.2, 171.8, 170.7, 158.5, 80.7, 78.5, 76.8, 67.5, 66.9, 53.7, 47.7, 47.4, 43.1, 42.1, 34.8, 28.7, 28.1, 26.7, 26.1, 18.1, 18.0. MS (MALDI$^+$) *m/z (%):* found *1169.6 (100),* calculated for $[C_{50}H_{78}N_6O_{24},Na]^+$ *1169.5.* FTIR (ν_{max}/cm^{-1}, ATR): 3479 - 3192 (N-H st and ≡C-H st), 2980 and 2937 (C-H st), 1745 (C=O st ester), 1693 (C=O st carbamate), 1519 (N-H δ), 1367 (C-N st), 1283 and 1250 (COO st), 1153 (O-C-C st).

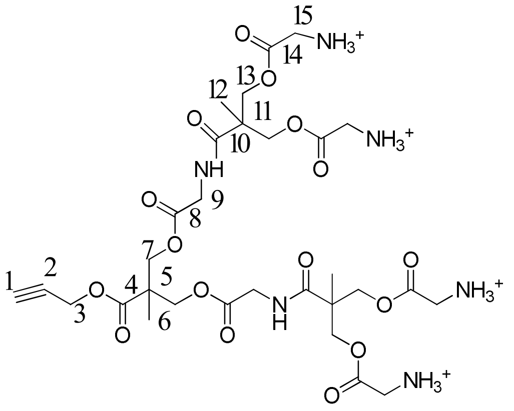

Compound 7. The t-Boc groups of compound **6** were cleaved following the deprotection method B described in section 2.1.3 in the manuscript. Compound **6** (1.43 g, 1.24 mmol, 1.00 eq.). Reaction time: 3 hours. A white powder was obtained (1.19 g, 80 %). ^{1}H NMR (400 MHz, CD_3OD) δ (ppm): 4.77 (d, J = 2.5 Hz, 2H, H-3), [4.48 – 4.37] (m, 8H, H-13), 4.32 (s, 4H, H-7), 3.95 (s, 4H, H-9), 3.91 (s, 8H, H-15), 2.99 (t, J = 2.4 Hz, 1H, H-1), 1.36 (s, 6H, H-12), 1.29 (s, 3H, H-6). ^{13}C NMR (100 MHz, CD_3OD) δ (ppm): [175.0 - 168.3], 163.1, 162.8, 78.4, 76.8, 68.5, 67.9, 67.1, 53.7, 47.7, 47.4, 42.0, 41.0, 28.0, 17.8, 17.6. MS (MALDI$^+$) *m/z (%)*: found *747.6 (77)*, calculated for $[C_{30}H_{46}N_6O_{16},H]^+$ *747.3*. FTIR (ν_{max}/cm^{-1}, ATR): 3371 - 2547 (bs N-H$^+$ st), 1730 (C=O st ester), 1670 and 1637 (C=O st carbamate), 1537 (N-H$^+$ δ), 1413 (C-N st), 1190 and 1136 (O-C-C st).

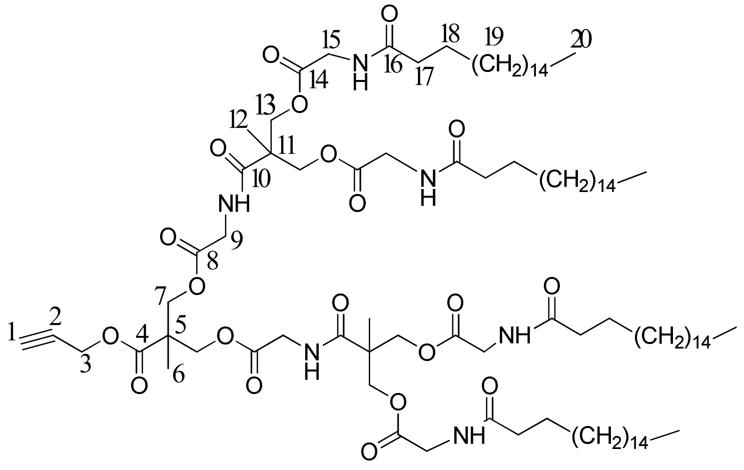

≡-[GMPA](C17)$_4$ Stearic acid (1.68 g, 5.91 mmol, 6.00 eq.), DMAP (0.19 g, 1.57 mmol, 1.60 eq.), HOBt (0.80 g, 5.91 mmol, 6.00 eq.) and DCC (1.22 g, 5.91 mmol, 6.00 eq.) were dissolved into dry DCM (45 mL). Compound **7** (1.18 g, 0.98 mmol, 1.00 eq.) and DMAP (0.48 g, 3.94 mmol, 4.00 eq.) were dissolved in anhydrous DMSO (5 mL). The final reaction mixture was stirred at room temperature under argon atmosphere for 48 hours. The white precipitate which appeared, DCU, was filtered off and the solvent was evaporated under vacuum. The crude product was dissolved into the minimal volume of DCM and purified by precipitation into cold acetone overnight. Finally, the precipitate was recovered by centrifugation at 3500 rpm for 5 minutes and was further purified by two consecutives silica gel columns (DCM: MeOH, 98: 2 and a gradient DCM: MeOH, 100: 0 to 98: 2). A yellow waxy solid was obtained (0.55 g, 31 %). ^{1}H NMR (400 MHz, $CDCl_3$) δ (ppm): 6.52 (bs, -NH), 4.74 (d, J = 2.4 Hz, 2H, H-3), [4.41-4.20] (m, 12H, H-7, H-13), 4.02 (d, J = 5.4 Hz, 8H, H-15), 3.97 (d, J = 5.6 Hz , 4H, H-9), 2.53 (t, J = 2.4 Hz, 1H, H-1), 2.24 (t, J = 7.7 Hz, 8H, H-17), 1.62 (m, 8H, H-18), 1.25 (s, 121H, H-6, H-12, H-19), 0.88 (t, J = 6.8 Hz, 12H, H-20). ^{13}C NMR (100 MHz, $CDCl_3$) δ (ppm): 174.2, 173.0, 171.9, 169.9, 169.5, 75.7, 66.8, 65.3, 52.9, 46.1, 41.5, 36.4, 32.1, [29.8-29.5], 25.7, 22.8, 18.1, 14.2. MS (MALDI$^+$) *m/z (%): found 1835.8 (100)*, calculated for $[C_{102}H_{182}N_6O_{20},Na]^+$ *1834.3*. FTIR (ν_{max}/cm^{-1}, ATR): 3301 (N-H st), 2916 and 2851 (C-H st), 1745 (C=O ester st), 1645 (C=O amide st), 1537 (N-H δ), 1468 (CH_2, CH_3 δ), 1200 (C-O st). Spectra are shown in figure S2.

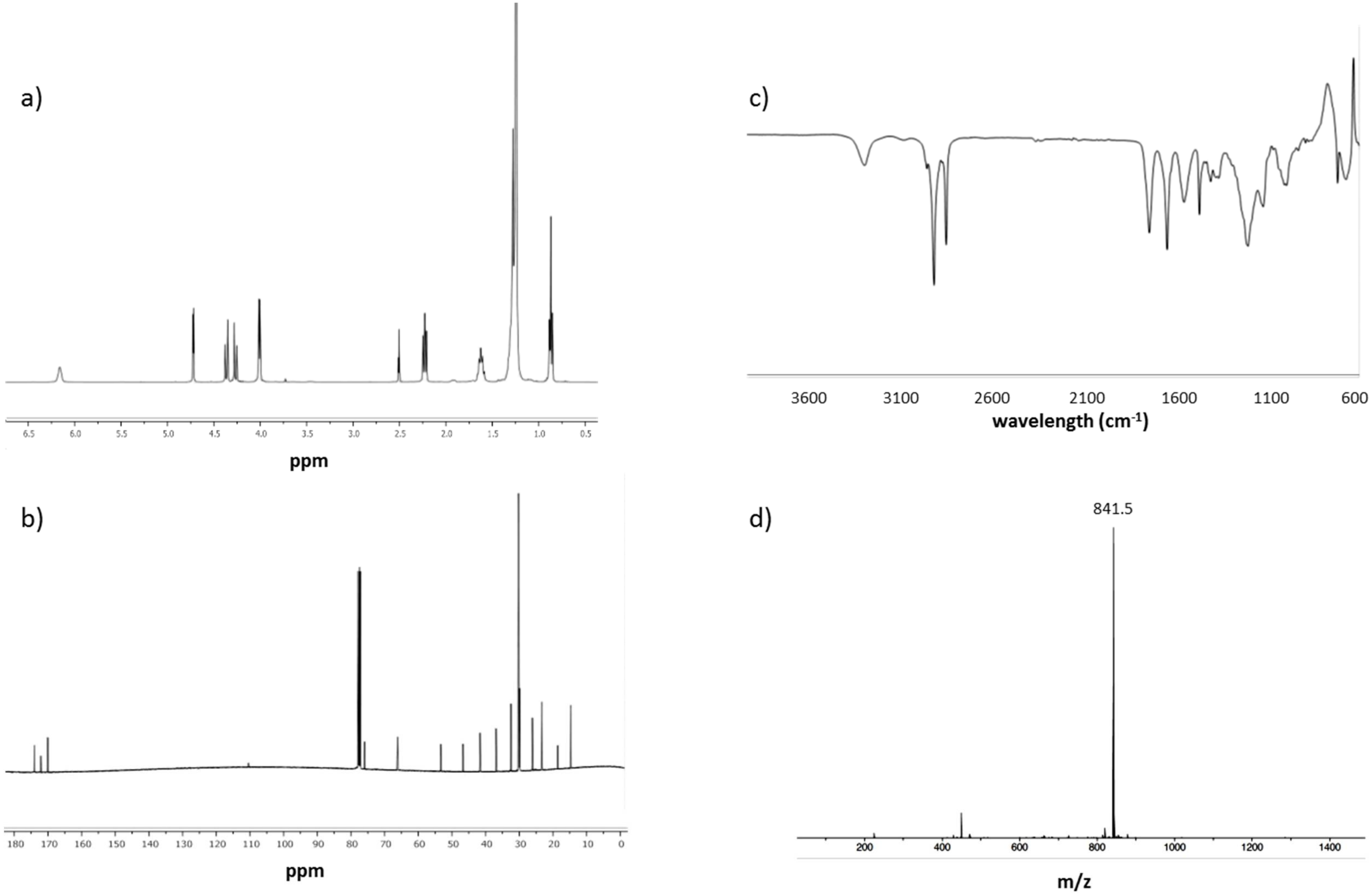

Figure S1. Chemical characterization of **≡-[GMPA](C17)$_2$**: a) ^{1}H NMR, b) ^{13}C NMR, c) FTIR spectrum in transmission mode and d) MS spectrum.

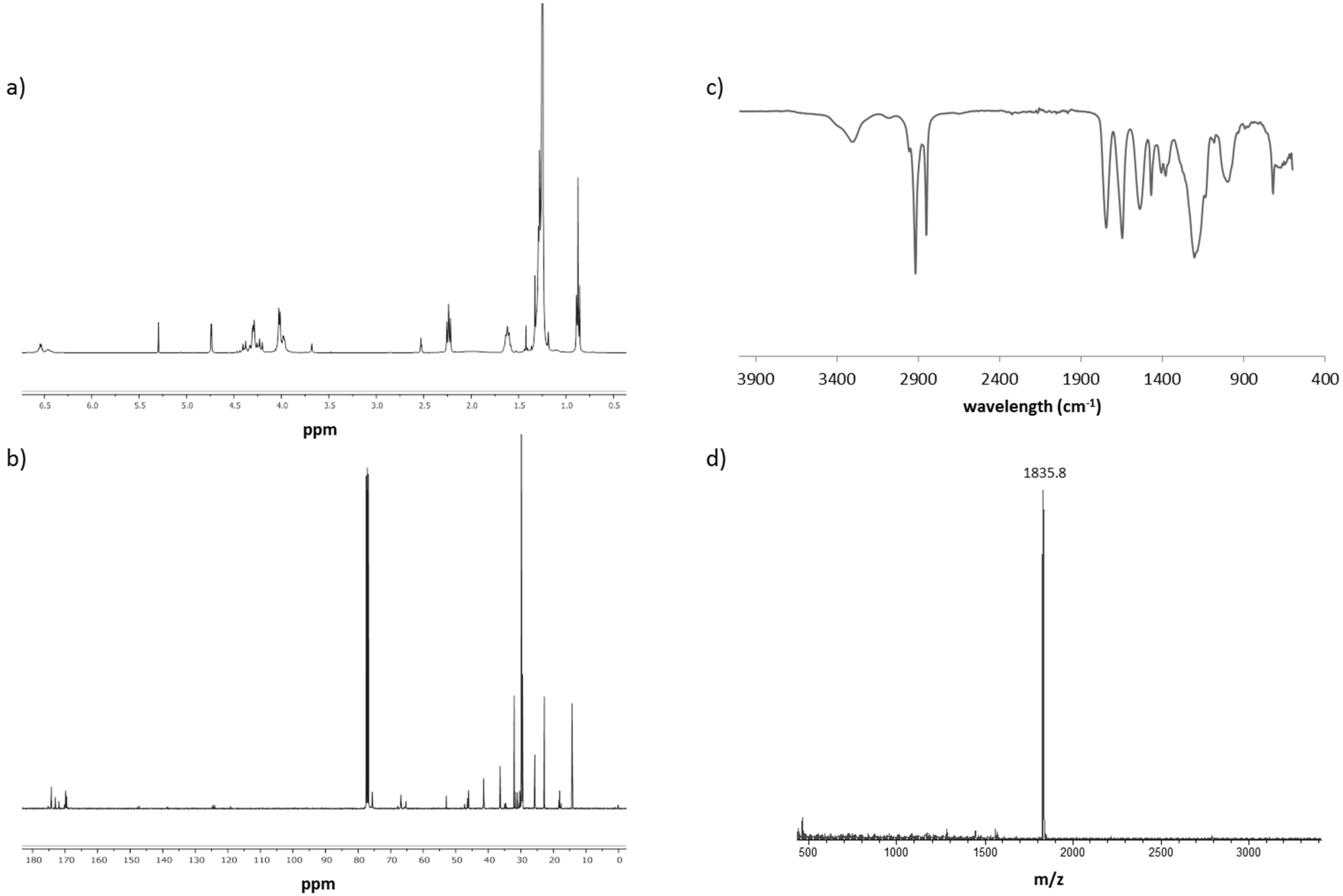

Figure S2. Chemical characterization of **≡-[GMPA](C17)$_4$**: a) ^{1}H NMR, b) ^{13}C NMR, c) FTIR spectrum in transmission mode and d) MS spectrum.

pharmaceutics

MDPI

2. Further characterization of dendrimer $(NH_3^+)_8$[GMPA]-[MPA](C17)$_4$

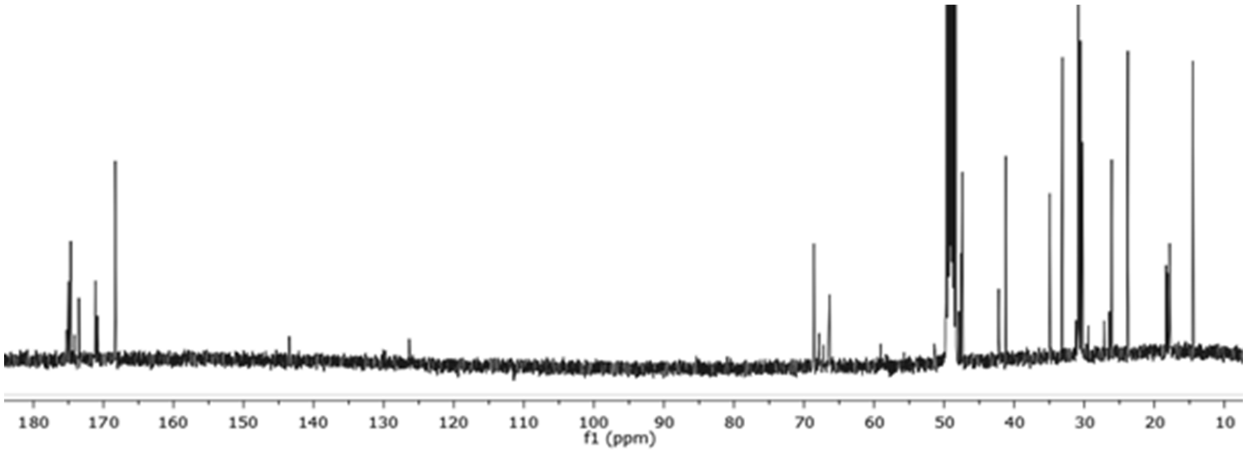

Figure S3. Full ^{13}C NMR spectrum of dendrimer $(NH_3^+)_8$[GMPA]-[MPA](C17)$_4$.

3. Critical aggregation concentration determination

In the presence of the Janus dendrimers and below the **Critical aggregation concentration (**CAC), nile red is located in water and presents weak fluorescence emission intensity. In contrast, above the CAC, nile red migrates to the lipophilic inner part of the dendrimer aggregates and presents acute fluorescence emission intensity (λ_{max} ≈ 635 nm).

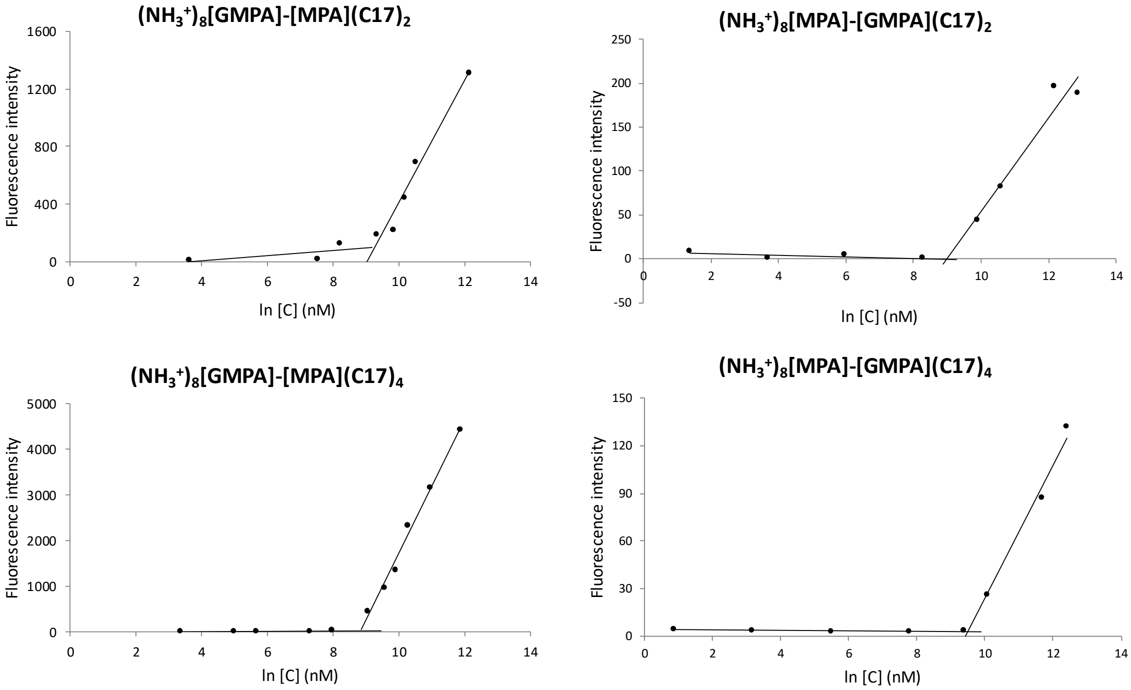

Figure S4. Determination of the CAC of all the Janus dendrimers by the nile red method.

pharmaceutics

4. Dynamic light scattering size determination

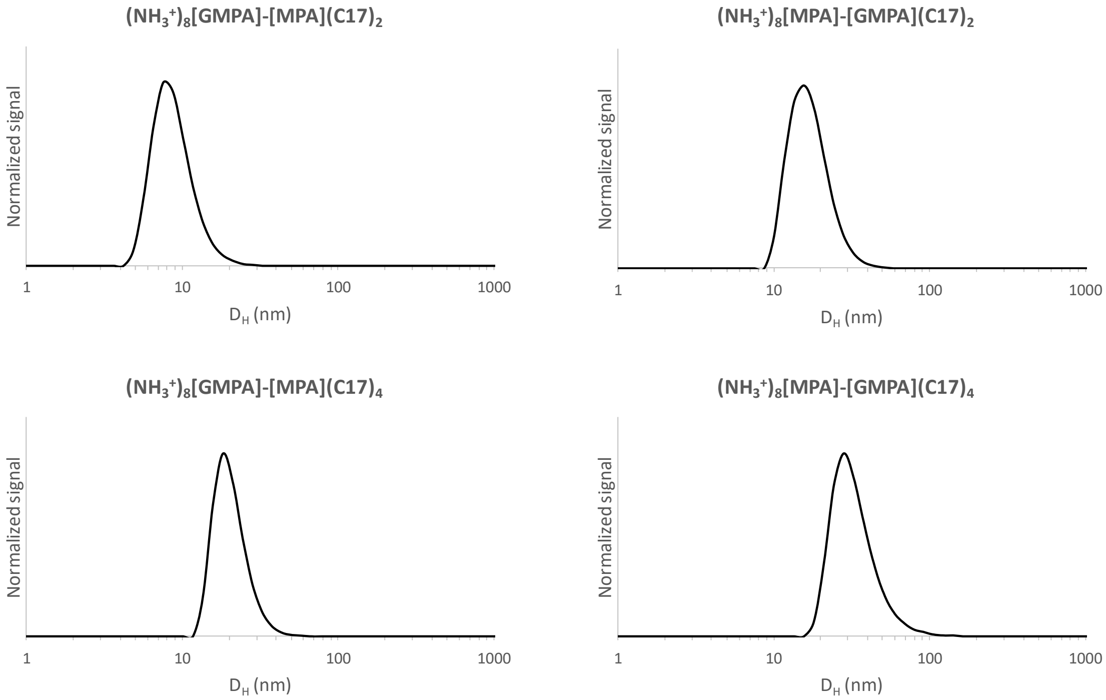

Figure S5. Size distribution measured by DLS.

5. Isothermal titration calorimetry experiments

$(NH_3^+)_8$[GMPA]-[MPA](C17)$_4$	$(NH_3^+)_8$[MPA]-[GMPA](C17)$_2$	$(NH_3^+)_8$[MPA]-[GMPA](C17)$_4$

a) IA

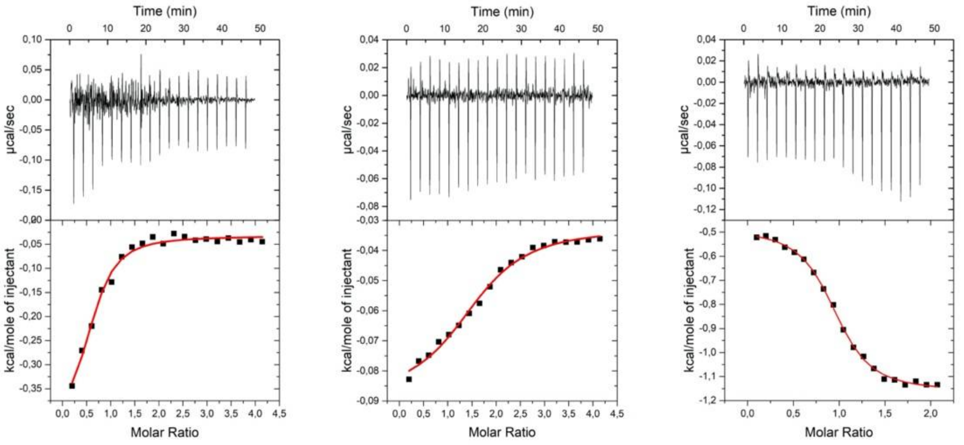

b) TRIAC

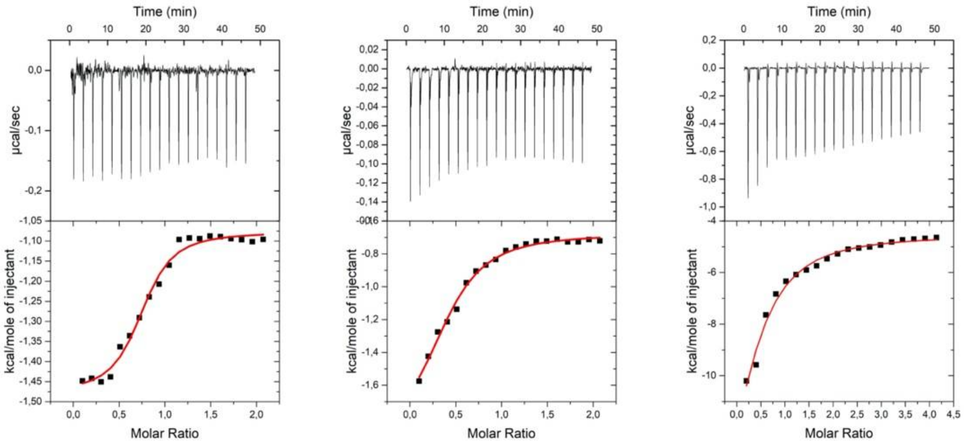

Figure S6. Interaction of drugs IA (a) and TRIAC (b) with **$(NH_3^+)_8$[GMPA]-[MPA](C17)$_4$, $(NH_3^+)_8$[MPA]-[GMPA](C17)$_2$** and **$(NH_3^+)_8$[MPA]-[GMPA](C17)$_4$** Janus dendrimer aggregates assessed by isothermal titration calorimetry (ITC). The upper plots in (a) and (b) show the thermogram (thermal power required to maintain a null temperature difference between sample and reference cells as a function of time) and the lower plots in A and B show the binding isotherm (ligand-normalized heat effect per injection as a function of the molar ratio, the quotient between the ligand and dendrimer concentrations in the cell). The fitting curve (in red) corresponds to the single ligand binding site model (continuous line).

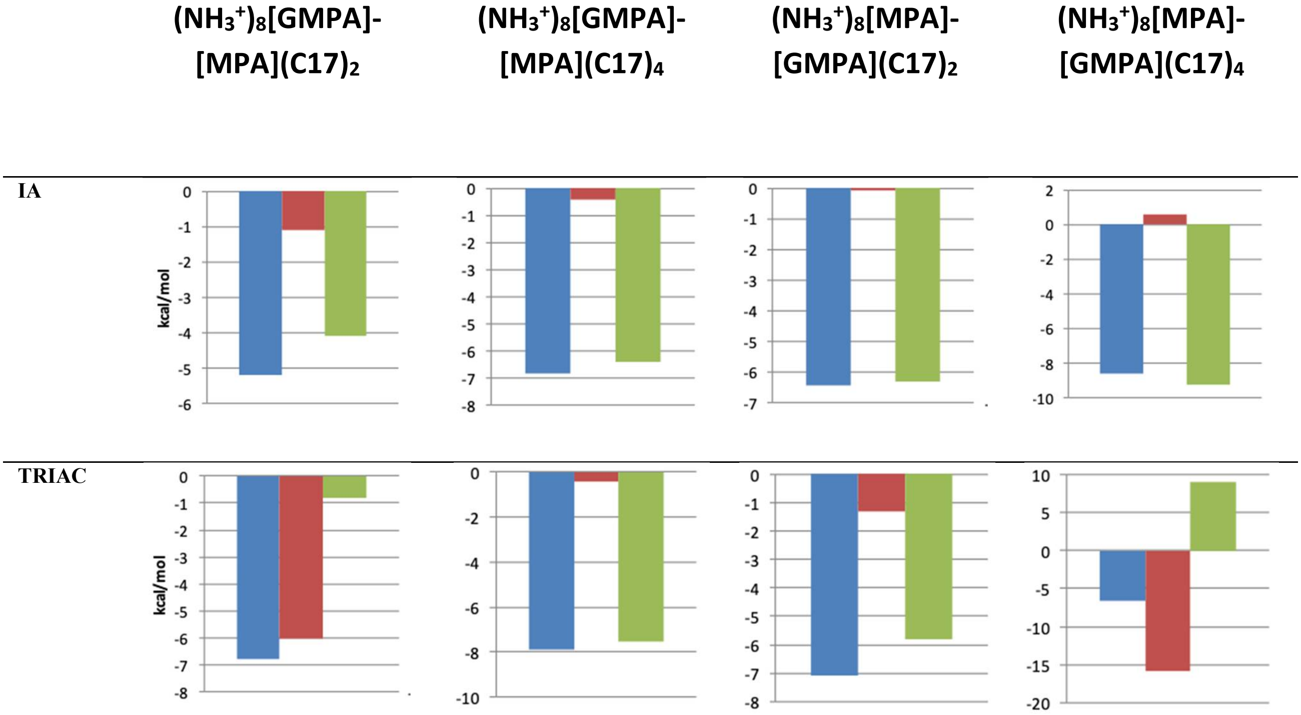

Figure S7. Graphical representation of thermodynamic parameters calculated from ITC assays for the interaction between Janus dendrimers and the compounds IA and TRIAC (extracted from Table 2). ΔG (blue bars), ΔH (red bars) and -T·ΔS (green bars) are expressed in kcal/mol.

6. Drug-loading

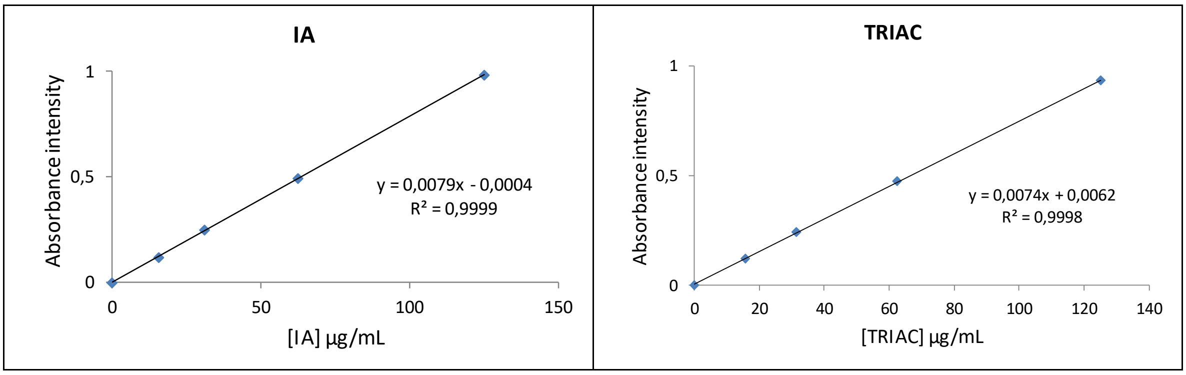

Figure S8. Calibration curves used for the determination of IA and TRIAC concentrations.

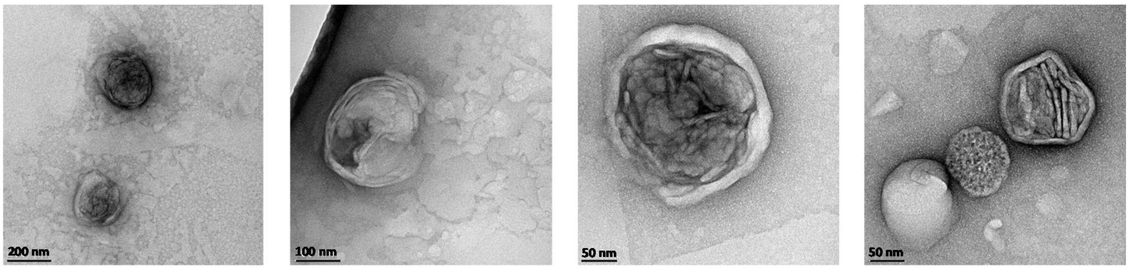

Figure S9. Additional TEM images of the **$(NH_3^+)_8$[GMPA]-[MPA](C17)$_4$** aggregates loaded with IA. Scale bar ranging from 200 to 50 nm.

7. Antiviral activity studies

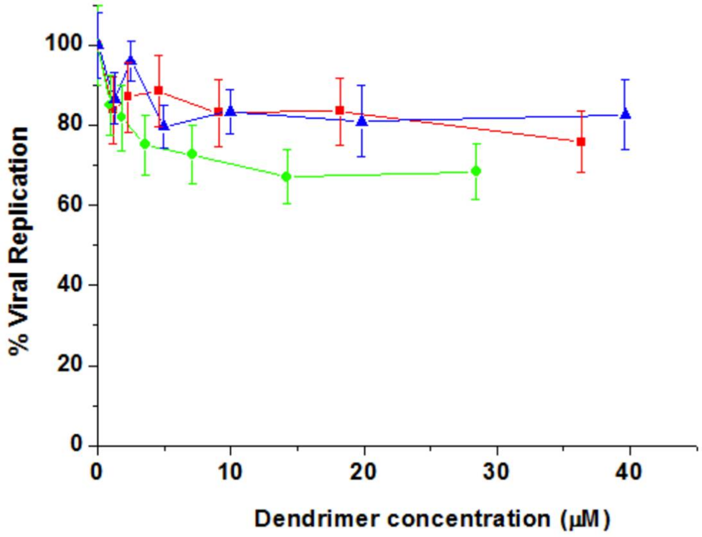

Figure S10. HCV viral replication of the Janus dendrimers in Huh 5-2 cell line. All the data are presented as the average ± standard deviation. **$(NH_3^+)_8$[GMPA]-[MPA](C17)$_2$** (Red Line) **$(NH_3^+)_8$[GMPA]-[MPA](C17)$_4$** (Green Line) and **$(NH_3^+)_8$[MPA]-[GMPA](C17)$_2$** (Blue Line).

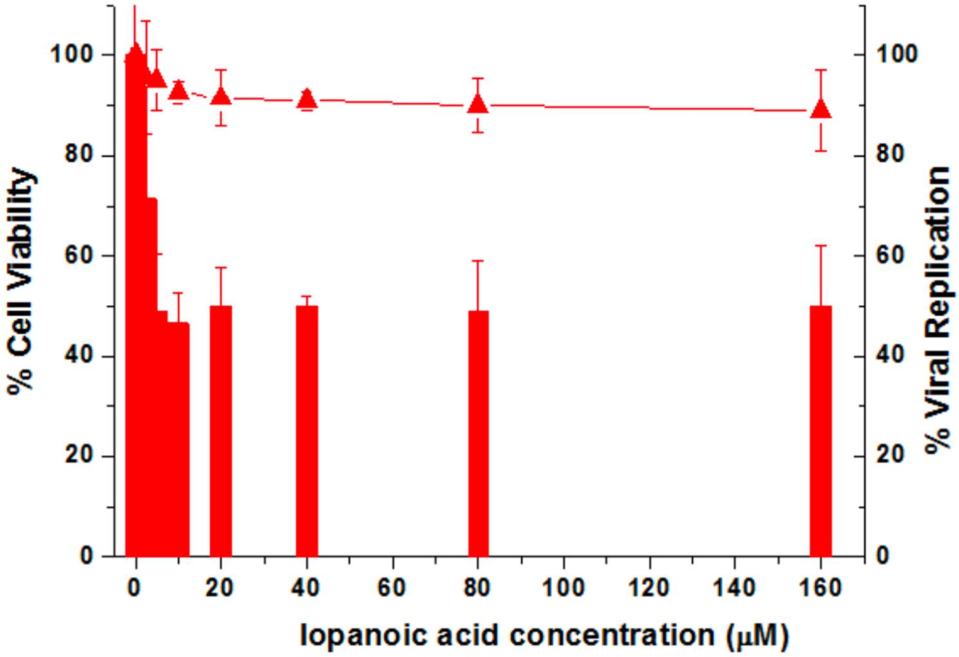

Figure S11. Cell viability (line) and HCV viral replication (bars) of **$(NH_3^+)_8$[GMPA]-[MPA](C17)$_2$/IA** in Huh 5-2 cell line including the highest concentration assayed, i.e. 160 µM. All the data are presented as the average ± standard deviation. .

8. Dendrimer aggregates labeling for cytometry assay

RhB(C17)$_2$, a rhodamine derivative, was used to label the **$(NH_3^+)_8$[GMPA]-[MPA](C17)$_4$** dendrimer aggregates (Figure S9). The synthesis of this derivative has been previously described by us [2]

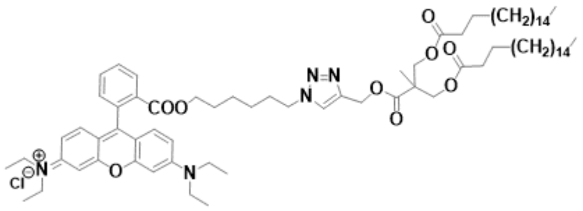

Figure S12. Chemical structure of RhB(C17)$_2$.

To label the dendrimer aggregates, the oil-in-water technique was employed. Firstly, the Janus dendrimer **$(NH_3^+)_8$[GMPA]-[MPA](C17)$_4$** was dissolved at a concentration of 284 μM (0.9 mg/mL) in dichloromethane. Accurate amount of RhB(C17)$_2$ was added to the previously solution to reach the ratio of 0.4 mol of RhB(C17)$_2$ per 1 mol of dendrimer (0.15 mg/mL). Secondly, a volume of water equal to that of dichloromethane was added to the solution. The biphasic mixtures were vigorously stirred at room temperature under ventilation until all dichloromethane was evaporated giving an aqueous dendrimer aggregates solution labelled with rhodamine derivative with a final concentration of 284 μM of **$(NH_3^+)_8$[GMPA]-[MPA](C17)$_4$** and 114 μM of Rh(C17)$_2$. As any precipitate was observed in the final labelled aqueous dendrimer aggregates solution, it was assumed that all the Rh(C17)$_2$ was integrated within the dendrimer aggregates. The labelling process was repeated twice, leading to the same result.

9. References

1 Lancelot, A.; González-Pastor, R.; Clavería-Gimeno, R.; Romero, P.; Abian, O.; Martín-Duque, P.; Serrano, J. L.; Sierra, T. Cationic Poly(Ester Amide) Dendrimers: Alluring Materials for Biomedical Applications. *J. Mater. Chem. B* **2018**, *6*, 3956–3968. https://doi.org/10.1039/C8TB00639C.

2 Jiménez-Pardo, I.; González-Pastor, R.; Lancelot, A.; Claveria-Gimeno, R.; Velázquez-Campoy, A.; Abian, O.; Ros, M. B.; Sierra, T., Shell Cross-Linked Polymeric Micelles as Camptothecin Nanocarriers for Anti-HCV Therapy. *Macromol. Biosci.* **2015,** *15*, 1381–1391. https://doi.org/10.1002/mabi.201500094.
